# RBCK1 is an endogenous inhibitor for triple negative breast cancer via hippo/YAP axis

**DOI:** 10.1186/s12964-022-00963-8

**Published:** 2022-10-24

**Authors:** Zhongbo Li, Peng Su, Yinlu Ding, Honglei Gao, Huijie Yang, Xin Li, Xiao Yang, Yan Xia, Chenmiao Zhang, Mingxi Fu, Dehai Wang, Ye Zhang, Shu Zhuo, Jian Zhu, Ting Zhuang

**Affiliations:** 1 Xinxiang Key Laboratory of Tumor Migration and Invasion Precision Medicine, School of Laboratory Medicine, Xinxiang Medical University, Xinxiang, 453003 Henan Province People’s Republic of China; 2 Department of Pathology, Shandong University Qilu Hospital, Cheeloo College of Medicine, Shandong University, Shandong, Shandong Province People’s Republic of China; 3 Department of General Surgery, The Second Hospital, Cheeloo College of Medicine, Shandong University, Shandong, Shandong Province People’s Republic of China; 4 Department of General Surgery, Weifang People’s Hospital, Shandong, Shandong Province People’s Republic of China; 5 Signet Therapeutics Inc., Shenzhen, 518017 People’s Republic of China

**Keywords:** RBCK1, YAP, Breast cancer, Ubiquitin

## Abstract

**Background:**

Triple negative breast cancer (TNBC) is one of the most lethal breast cancer subtypes. Due to a lack of effective therapeutic targets, chemotherapy is still the main medical treatment for TNBC patients. Thus, it is important and necessary to find new therapeutic targets for TNBC. Recent genomic studies implicated the Hippo / Yap signal is over activated in TNBC, manifesting it plays a key role in TNBC carcinogenesis and cancer progression. RBCK1 was firstly identified as an important component for linear ubiquitin assembly complex (LUBAC) and facilitates NFKB signaling in immune response. Further studies showed RBCK1 also facilitated luminal type breast cancer growth and endocrine resistance via trans-activation estrogen receptor alpha.

**Methods:**

RBCK1 and YAP protein expression levels were measured by western blotting, while the mRNA levels of YAP target genes were measured by RT–PCR. RNA sequencing data were analyzed by Ingenuity Pathway Analysis. Identification of Hippo signaling activity was accomplished with luciferase assays, RT–PCR and western blotting. Protein stability assays and ubiquitin assays were used to detect YAP protein degradation. Ubiquitin-based immunoprecipitation assays were used to detect the specific ubiquitination modification on the YAP protein.

**Results:**

In our current study, our data revealed an opposite function for RBCK1 in TNBC progression. RBCK1 over-expression inhibited TNBC cell progression in vitro and in vivo, while RBCK1 depletion promoted TNBC cell invasion. The whole genomic expression profiling showed that RBCK1 depletion activated Hippo/YAP axis. RBCK1 depletion increased YAP protein level and Hippo target gene expression in TNBC. The molecular biology studies confirmed that RBCK1 could bind to YAP protein and enhance the stability of YAP protein by promoting YAP K48-linked poly-ubiquitination at several YAP lysine sites (K76, K204 and K321).

**Conclusion:**

Our study revealed the multi-faced RBCK1 function in different subtypes of breast cancer patients and a promising therapeutic target for TNBC treatment.

**Video abstract**

**Supplementary Information:**

The online version contains supplementary material available at 10.1186/s12964-022-00963-8.

## Background

Breast cancer is the leading cause for female malignancies and ranks NO.2 in women cancer mortality [1]. Among all subtypes of breast malignancies, TNBC is the most aggressive subtype and the worst in prognosis [2]. Since a lack of effective targets, such as estrogen Receptor and Human Epidermal Growth Factor Receptor 2, which could be blocked by tamoxifen and Herceptin, the treatment of TNBC depends on chemotherapy [3]. In addition, the definition of TNBC is an exclusive conception, which includes the non-ER and non-HER2 expression types [4]. Thus, it includes these types of unclassified breast cancers, making it a big challenge to identify certain molecular markers for targeted therapy in TBNC. Recent genomic studies revealed that TNBC included higher genomic abnormalities, such as gene mutations and gene amplifications [5, 6]. Several oncogenes, such as YAP, were amplified in TNBC [7, 8]. Based on the fact of high heterogeneous of TNBC, it is urgent and necessary to identify novel therapeutic targets for breast cancer treatment.

Quite a few studies have revealed the critical function of Hippo signaling in TNBC progression [9–12]. The normal function of Hippo signaling is important in tissue regeneration, organ size control and cancer prevention [13]. However, the dys-function of Hippo signaling could commonly be observed in several human cancers, including gastric cancer and TNBC [14, 15]. The activation of Hippo signaling causes the phosphorylation of LATS by MST1/2. Subsequently, LATS promotes the phosphorylation of YAP, which inhibits the YAP translocation into the nuclear for the target gene expression, such as CTGF and CYR61. On the other hand, phosphorylated YAP is retained into the cytosol and leads to the protein degradation [16–18]. In human malignancies, the Hippo/YAP axis is always activated. For example, YAP protein is over-expressed in several human cancers, including TNBC[19–22]. Interestingly, depletion YAP expression or blockage of YAP function in TNBC causes cancer growth inhibition, which means YAP could be a therapeutic target for TNBC treatment [23, 24]. Although several studies attempted to identify effective inhibitors, pharmaceutically targeting YAP function is still premature in clinical application.

Although the activity of Hippo signaling is tightly controlled through the phosphorylation cascade, it is mysterious that YAP protein is over-active, even the Hippo signaling is still functional [25]. Besides phosphorylation, several other post-translational modifications were found to play critical roles in modulation YAP protein stability and function[23, 26, 27]. Our previous studies revealed a few E3 ubiquitin ligases, which modulated YAP protein ubiquitination via different mechanisms [10, 11, 23, 26, 28]. RBCK1 (RANBP2-type and C3HC4-type zinc finger-containing 1) belongs to the RING finger family and is composed of 510 amino acids [29]. Previous studies revealed that RBCK1 mainly exerted its function via its ubiquitin ligase function and facilitated luminal type breast cancer cell progression and tamoxifen resistance via estrogen signaling[30]. Based on our expertise in ubiquitin ligases, we further investigated RBCK1 function in TNBC subtype, which revealed the tumor suppressor roles in TNBC through inhibition Hippo/YAP axis. Our study implicated the muti-faced function of RBCK1 in different subtype of breast cancers and an interesting therapeutic target for TNBC treatments.

## Materials and methods

### Cell culture

MDA-MB-231, BT549, and HEK293 cells were obtained from the American Type Culture Collection (ATCC). MDA-MB-231 and HEK293 cells were cultured in Dulbecco’s modified Eagle’s medium containing 4.5 g/L glucose and 4 mM L-glutamine (DMEM, 41,965, Life Technologies) and supplemented with 10% fetal bovine serum (FBS, 10,270, Life Technologies). BT549 cells were cultured in RPMI-1640 medium (42,401, Life Technologies) supplemented with 2 mM L-glutamine (25,030, Life Technologies) and 10% FBS. All cell lines were identified by cell line authentication. Cell line authentication was performed via short tandem repeat (STR) profiling in a PowerPlex 21 system. We found that the STR data of MDA-MB-231, BT549 and HEK293 cell lines were consistent with STR data in ATCC (Additional file 1).

### RNA isolation and quantitative real-time PCR (qRT-PCR)

We used RNeasy plus mini kits to extract the total RNA according to the protocol (Tiangen). Reverse transcription was performed using a RevertAid First Strand cDNA Synthesis Kit (Thermo, Lithuania). qRT-PCR was carried out using GoTaq® qPCR Master Mix (Promega, USA) in a 7500 Fast Real-Time PCR System (Applied Biosystems, Singapore). The real time PCR reaction contains two steps. The annealing temperature is 60 °C. The 36B4 gene was used as the internal control. The primer sequences were shown here. RBCK1: F: tgc tca gat gca cac cgt c, R: caa gac tgg tgg gaa gcc ata. 36B4: F: ggc gac ctg gaa gtc caa ct; R: cca tca gca cca cag cct tc. CTGF: F: ctc gcg gct tac cga ctg; R: ggc tct gct tct cta gcc tg. CYR61: F: agc agc ctg aaa aag ggc aa; R: agc ctg tag aag gga aac gc. The specificity of all primer pairs was checked with melting curve analysis.

### Western blot analysis

The standard Western blotting program is used to detect cells In Western blot analysis, the following antibodies were used to analyze protein expression: anti-HA (901514, Biolegend, 1:1000), anti-Myc (AB32, Abcam, 1:1000), anti-Flag(Ab49763, Abcam,1:1000), anti-Actin (3700, Cell Signaling Technology, 1:1000), anti-RBCK1 (26367-1-AP, Proteintech, 1:1000), anti-YAP (SC-101199,, Santa Cruz, 1:1000), anti-Tubulin (11224-1-AP, Proteintech, 1:1000), anti-Histone-H3 (17168-1-AP, Proteintech, 1:1000). The protein signal was detected by ECL kit (Millipore Co., Billerica, Massachusetts, USA).

### Plasmids and siRNA

The Flag-RBCK1 plasmid was acquired from Origene (RC229128). The RBCK1 deletion constructs were purchased from HANBIO Biological (Shanghai, China). The HA-Ub, HA-K48, and HA-K63 plasmids were used in previous study[31]. Plasmids were transfected with Lipofectamine 2000 (1,662,298, Invitrogen). The RBCK1 siRNA sequences were: siRNA#1:GUGCCUACCUCUAUCUGCUdTdT: AGCAGAUAGAGGUA GGCACdTdT and siRNA#2: GCCUUCAGCUACCAUUGCAdTdT; UGCAAUGGUAGC UGAAGGCdTdT. The negative control siRNA sequences were: UUCUCCGAACGUGUC ACGUTT; ACGUGACACGUUCGGAGAATT.

### Quantification of cell viability

The siRBCK1 or siControl were transfected into MDA-MB-231 and BT549 cells in 24 well plates. After 24 h of transfection, the cells were counted, and 4000 cells were inoculated into 96 well plates. We measure the relative cell viability at the specified time point. The number of cells was determined using CCK8 cell proliferation reagent by measuring the absorbance at 450 nm.

### Trans-well assay

We used a new two compartment plates to check the capacity of cell migration and invasion. For migration test, cells in serum-free medium were inoculated into the upper chambers. For the invasion test, the upper chambers were coated with matrigel (BD Biocoat, USA) After 12 h, we carefully take out the cells, fixed the cells invading the cell membrane, and fill with Crystal Violet Staining solution. The cells were then observed and taken photos under a microscope and counted with ImageJ.

### Flow cytometric analyses

For apoptosis analysis, MDA-MB-231 and BT549 cells were transfected with siRBCK1 or siControl. 24 h after transfection, the cells were stained with propidium iodide and annexin V. The fluorescence intensity was measured by BD LSR flow cytometry. For (CD44 / CD24) cell ratio analysis, MDA-MB-231 and BT549 cells were transfected with siRBCK1 or siControl. 24 h after transfection, the breast cancer cells in logarithmic growth phase were digested by 0.25% trypsin and washed for 3 times by PBS, then resuspension in 100ul PBS, and then stained with anti-CD44-PE and anti-CD24-FITC. Then wash the sample with PBS for 3 times, and finally at 200 μ L resuspend in PBS. Flow cytometry analysis was performed on a BD AccuriTM C6 Flow Cytometer (BD Bioscience). According to the percentage of CD44 and CD24 positive subsets in flow cytometry, calculate the expression rates of CD44 and CD24 (CD44/CD24) in different subtypes of breast cancer cell lines.

### Immunoprecipitation

Immunoprecipitation was performed as described in the previous study [32]. The total lysate of MDA-MB-231 cells was precleared with rabbit IgG for 2 h, and then immunoprecipitation was carried out at night with anti-RBCK1 antibody (26367-1-ap, Proteintech, 1:1000) or anti-YAP antibody (SC-101199, Santa Cruz, 1 / 400). The bounded protein was detected through anti-YAP antibody (SC-101199, Santa Cruz; 1/2000) or anti-RBCK1 antibody (26367-1-AP, Proteintech, 1:2000). In the overexpression experiment, HEK293 cells were cotransfected with 5 μg of Flag-RBCK1 plasmid (full-length RBCK1 or domain deletion mutants) and 5 μg of YAP plasmid. Cell lysates were precleared with IgG and then incubated with an anti-YAP (SC-101199, Santa Cruz) antibod. The binding protein was analyzed by western blotting with an anti-Flag antibody (Ab49763, Abcam). Therefore, in a 10 cm dish, Myc-YAP plasmid (full-length Yap or domain deletion mutant) was mixed with 5 μg Flag-RBCK1 plasmid was co transfected. Cell lysates were precleared with IgG, then incubated with an anti-Myc (AB32, Abcam) antibody, while rabbit IgG was used as the negative control. Binding proteins were analyzed by western blotting.

### Protein stability assays

Approximately 10^5^ MDA-MB-231 and BT549 cells were inoculated into 24 well plates and transfected with 50 μM RBCK1 siRNA or siControl transfection. 48 h later, the cells after 100 μM cycloheximide (C7698, Sigma) treatment was treated at indicated times. For HEK293 cells, 10^5^ cells were inoculated into 24 well plates and transfected with 0.5 μg Flag-RBCK1 or Flag-vector transfection. 48 h later, the cells after 100 μM cycloheximide (C7698, Sigma) treatment was treated at special time points. The sample was subjected to western blotting to evaluate YAP level.

### In vivo tumorigenesis essay

In the tumorigenic experiment in vivo, we used female BALB/c nude mice aged 5 weeks in each set of groups. 3X10^6^ MDA-MB-231 cells were injected subcutaneously into each mouse. Tumor formation was tested about 5 weeks. The calculation formula of tumor volume is: tumor volume = length × width2 /2.

### Analysis of protein ubiquitination

Transfect 1.5 µg YAP plasmid and 1.5 µg Flag-RBCK1 or Flag-tag into HEK293T. 48 h later, cells were treated with 10 µM MG132 (474,787, Sigma) for 6 h, then the ubiquitination level of Myc-YAP was checked by IP with anti-Myc antibody, and then western blot analysis was performed with an anti-HA antibody (901,514, Biolegend, 1:2000).

### Immunofluorescence assay

MDA-MB-231 cells were fixed with 4% in PBS paraformaldehyde for 10 min, infiltrated with 0.2% Triton X-100 for 5 min, and blocked with 5% BSA in PBS for 1 h. Mouse anti-RBCK1 (SC-365523, Santa Cruz, 1:100) antibody and rabbit anti-YAP monoclonal antibody (13584-1-AP, Proteintech, 1:100) were used as primary antibodies, followed by Alexa Fluor 647-conjugated (Invitrogen) anti-rabbit and FITC-conjugated anti-mouse antibodies (Jackson ImmunoResearch, West Grove, PA) as secondary antibodies. As a negative control, the sample was incubated with the secondary antibodies without the primary antibody incubation step. The images were obtained under conditions satisfying the Nyquist criterion by using a Nikon A + laser scanning confocal microscope system with a 60X oil NA1.4 objective lens and pinhole size of 1.0 Airy unit. The acquired images were used to further process and assemble the collected ImageJ.

### Poly-ubiquitination assay

In order to directly detect K48 ubiquitinated and total ubiquitinated YAP enriched in cell extracts, K48 ubiquitinated, K63 ubiquitinated or UB plasmids, Flag-RBCK1 plasmids and Myc-YAP or Myc-Vectors were transfected into HEK293 cells. 24 h later, the cells were treated with 20 μM MG132 for 6 h. Then extract the overall protein and use 40 μL protein A (SC-2001, Santa Cruz) pre clarified the lysate for 4 h. The supernatant was collected and we use western blot to detect the immunoprecipitation with an anti-YAP antibody. Total polyubiquitinated YAP or K48/K63-polyubiquitinated YAP was detected by western blot with an anti-HA antibody.

### Wound healing assay

The cells were inoculated into a 12-well petri dishes containing 1% FBS. When the cells were 100% fused, we scratched it with the tip of the yellow pipette. The wound gap was detected at the appoint time point and standardized with the earliest time point. Formula for calculating wound healing recovery: [1—(Wound width at a given time/wound width at t = 0)] × 100%.

### Luciferase assay

The luciferase activity of TEAD was checked by using the Dual-Luciferase Reporter kit (Promega). Transfected with TEAD luciferase reporter Cells were collected, and luciferase activity was measured.

### Publicly available clinical data analysis

The gene expression data for 1211 TCGA breast cancer patients were obtained from the webpage (http://gepia.cancer-pku.cn/index.html). GEPIA online software shows the expression difference of RBCK1 mRNA level between normal breast tissue and different stages of breast cancer. KMPLOT online analysis database shows the progression-free survival (PFS) survival data of YAP and RBCK1 (https://kmplot.com). The gene affy ID was 213342_at for YAP and 207713_at for RBCK1.

### Clinical breast tumor samples

Qilu Hospital, Shandong University, provided 40 samples of triple negative breast cancer. Check the ER, PR and HER2 status of all these samples. The immunohistochemistry of RBCK1 and YAP was detected according to the standard method. Pathologists examined the IHC results of RBCK1 and YAP. IHC analysis was performed using rabbit anti-RBCK1 polyclonal antibody (26367-1-AP, Proteintech) and mouse anti-YAP monoclonal antibody (SC-101199, Santa Cruz). The size of FFPE slice was prepared in 4 μm. Two independent certified pathologists checked the result of YAP and RBCK1 staining.

### RNA sequencing and data analysis

Global gene expression analysis (siControl and siRBCK1) is based on the RNA sequencing platform of Beijing Genome Institute (BGI). RNA sequence data were stored in the gene expression comprehensive database (GEO) database (Assessing number: GSE195712). Differentially expressed genes were analyzed by ingenuity pathway analysis (IPA) (*P* < 0.01 and fold change > 2) For gene set enrichment analysis of RNA SEQ data, gene sets of Conserved Hippo Signature was used and downloaded from Molecular Signatures Database v7.4, GSEA was implemented using the GSEA 4.1.0 software, with default parameters. The volcanic map was generated by the "ggplot2" software package in R (threshold *P* < 0.05, fold change > 1.5).

### Statistical analysis

Statistical analysis does not use specific statistical tests to determine the sample size in advance. Statistical analysis was performed using GraphPad Prism 7 software or SPSS version 23.0. Data were expressed as the mean ± S.E.M. values. The difference between the two groups was assessed by Student t-test. The survival analysis used Kaplan–Meier method and log rank test. the difference was considered statistically significant. when *P* < 0.05 (*P < 0.01; **P < 0.001).

## Results

### RBCK1 depletion promotes cancer cell progression in TNBC

**Fig. 1:**
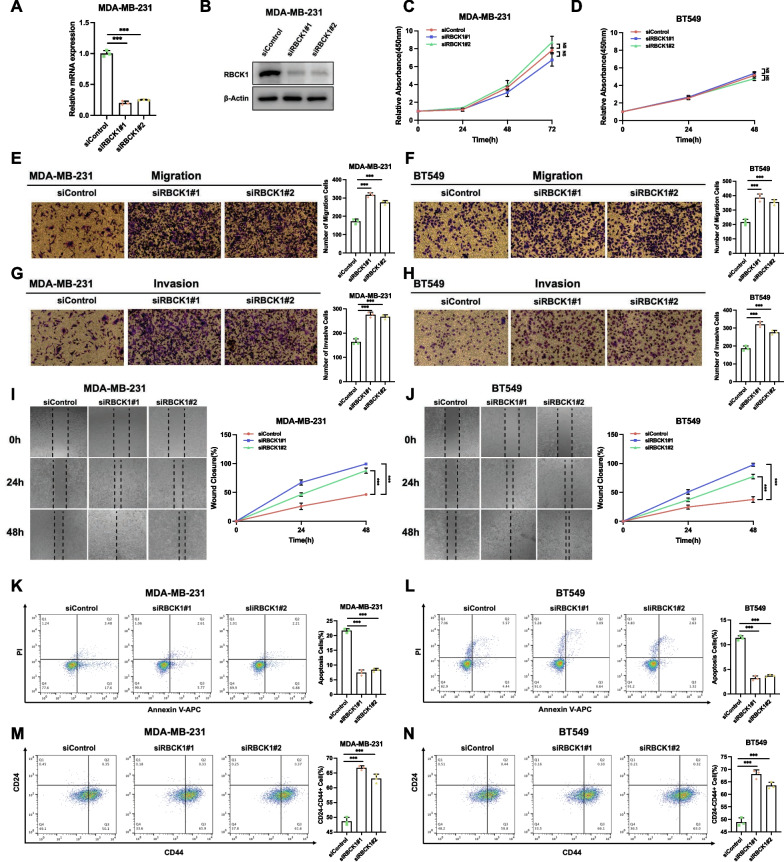
RBCK1 depletion promotes cancer cell progression in TNBC **A**–**B** RBCK1 deletion efficiency by two different siRNA oligonucleotides in breast cancer cell. *P < 0.05; **P < 0.01; ***P < 0.001 for RBCK1 mRNA level comparison. **C**–**D** RBCK1 depletion has no effect on TNBC cancer cell proliferation. Each group was analyzed three times. *P < 0.05; **P < 0.01; ***P < 0.001 for cell growth comparisons. **E**–**F** RBCK1 deletion promotes the migration of cell in TNBC.MDA-MB-231 and BT549 cells were transfected with siControl or siRBCK1. Calculate the number of cells and the number data showed as ± SD. *P < 0.05; **P < 0.01; ***P < 0.001 for cell comparisons. **G**–**H** RBCK1 depletion promotes the invasion of cell in TNBC.MDA-MB-231 and BT549 cells were transfected with siControl or siRBCK1. Calculate the number of cells and the number data showed as ± SD. *P < 0.05; **P < 0.01; ***P < 0.001 for cell comparisons. **I**–**J** Wound healing experiment of TNBC cells was transfected with siRBCK1 or siControl. Wound closure was quantified at a specified point in time. *P < 0.05; ** P < 0.01; ***P < 0.001 for comparisons. **K**–**L** RBCK1 depletion inhibited apoptosis in TNBC cells. MDA-MB-231 and BT549 cells were transfected with siControl or siRBCK1. Each group was analyzed three times. *P < 0.05; **P < 0.01; ***P < 0.001 for comparisons. **M**–**N** RBCK1 depletion increased the expression of CD24-CD44 + in TNBC cells. MDA-MB-231 and BT549 cells were transfected with siControl or siRBCK1. Each group was analyzed in three times. *P < 0.05; ** *P* < 0.01; ***P < 0.001 for comparisons

We firstly examined the silence efficiency of RBCK1 in TNBC cells, in which the QPCR and western blot data showed RBCK1 could be dramatically depleted in MDA-MB-231 cells (Fig. 1A,B). The CCK8 assay showed that RBCK1 depletion did not affect cell proliferation speed in MDA-MB-231 cells and BT549 cells (Fig. 1C,D). In trans-well assays with permeable filter and basement membrane, TNBC cell invasion and migration could be boosted by RBCK1 deletion (Fig. 1E–H). The wound healing experiments confirmed similar results that in both MDA-MB-231 and BT549 cell models, RBCK1 deficient cells had faster wound closure speed than wild-type cells (Fig. 1I,J). Besides, the proportion of apoptotic cells in MDA-MB-231 and BT549 cells could be decreased because of RBCK1 deficiency by the PI/Annexin V double staining combined with FACS analysis (Fig. 1K,L). Since several studies have shown that the stemness of TNBC (CD44 + /CD24−) is also an important property for cancer progression. We examined the effect of RBCK1 depletion in TNBC stemness, in which the data showed that RBCK1 depletion could further enhance the property of stemness in MDA-MB-231 and BT549 cells (Fig. 1M,N).

### RBCK1 overexpression inhibited TNBC cell progression in vitro and in vivo

**Fig. 2:**
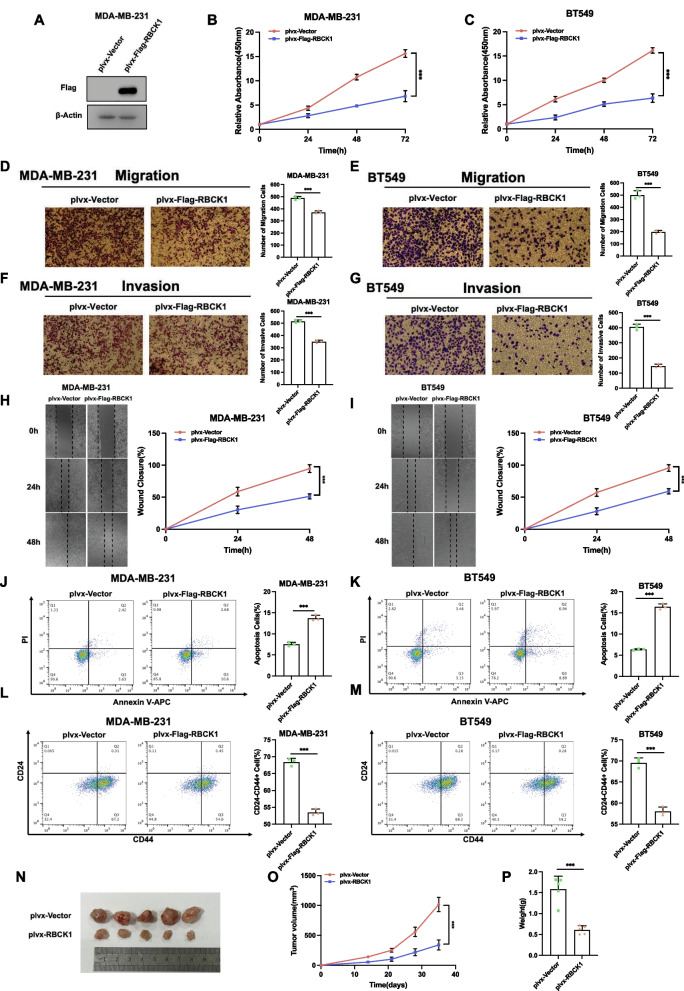
RBCK1 overexpression inhibited TNBC cell progression in vitro and in vivo. **A** RBCK1 over-expression efficiency by plasmids. We overexpressed RBCK1 in TNBC cells via lenti-virus infect system. The level of Flag-RBCK1 protein was detected by western blot. β-Actin was used as the internal reference. **B**–**C** Overexpression of RBCK1 inhibits the proliferation of TNBC cells. The metabolic activity of cells at the specified time points after transfection was determined by CCK-8 analysis. Each group was analyzed three times. *P < 0.05; **P < 0.01; ***P < 0.001 for cell growth comparisons. **D**–**E** Overexpression of RBCK1 inhibited cell migration in TNBC cells. MDA-MB-231 and BT549 cells with stable expression of RBCK1 or empty vector. Calculate the number of cells and the number data showed as ± SD. *P < 0.05; **P < 0.01; ***P < 0.001 for cell comparisons. **F**–**G** Overexpression of RBCK1 inhibited cell invasion in TNBC cells. MDA-MB-231 and BT549 cells with stable expression of RBCK1 or empty vector. Calculate the number of cells and the number data showed as ± SD. *P < 0.05; ** *P* < 0.01; ***P < 0.001 for cell comparisons. **H**–**I** Wound healing experiment of MDA-MB-231 and BT549 cells with stable expression of RBCK1 or vector. Wound closure was quantified at a specified point in time. *P < 0.05; **P < 0.01; ***P < 0.001 for cell comparisons. **J**–**K** Overexpression of RBCK1 increased apoptosis in TNBC cells. MDA-MB-231 and BT549 cells with stable expression of RBCK1 or empty vector were stained with PI and Annexin V. Each group was analyzed three times. *P < 0.05; **P < 0.01; ***P < 0.001 for comparisons. **L**–**M** Overexpression of RBCK1 inhibited the expression of CD24-CD44 + in TNBC cells. MDA-MB-231 and BT549 cells with stable expression of RBCK1 or empty vector were stained with anti-CD44-FITC and anti-CD24-PE. Each group was analyzed in three times. *P < 0.05; **P < 0.01; ***P < 0.001 for comparisons. **N**–**P** Overexpression of RBCK1 inhibited the tumor growth of MDA-MB-231 cells in a xenograft model. Tumor growth photographs, curves and weights are shown in panels N, O and P, respectively. The data are showed as ± SD. *P < 0.05; **P < 0.01; ***P < 0.001 for cell comparisons

In order to confirm the phenotype, we overexpressed RBCK1 in TNBC cells via lenti-virus infect system. The exogenous expression of RBCK1 was confirmed in Fig. 2A. The CCK8 assay showed that increased RBCK1 expression could cause inhibited cell proliferation in MDA-MB-231 and BT549 cells (Fig. 2B,C). In trans-well assays with permeable filter and basement membrane, TNBC cell invasion and migration could be decreased by the overexpression of RBCK1 (Fig. 2D–G). The wound healing experiments confirmed similar results that in both MDA-MB-231 and BT549 cell models, RBCK1 over-expression cells had decreased wound closure speed (Fig. 2H,I). The PI/Annexin V double staining coupled with FACS analysis showed that RBCK1 overexpression could facilitate cellular apoptosis in MDA-MB-231 and BT549 cells (Fig. 2J,K). We also measured the cell stemness property via CD44 and CD24 markers, in which RBCK1 overexpression could decrease the proportion of CD44 + /CD24- cells (Fig. 2L,M). Then, we generated a model of stable RBCK1 overexpression in MDA-MB-231 cells and further established a xenograft mouse model to study the role of RBCK1 in vivo. Data confirmed that overexpression of rbck1 could reduced the growth rate of tumors in vivo (Fig. 2N–P).

### Clinical data analysis reveals the correlation between RBCK1 and Hippo signaling in TNBC

**Fig. 3:**
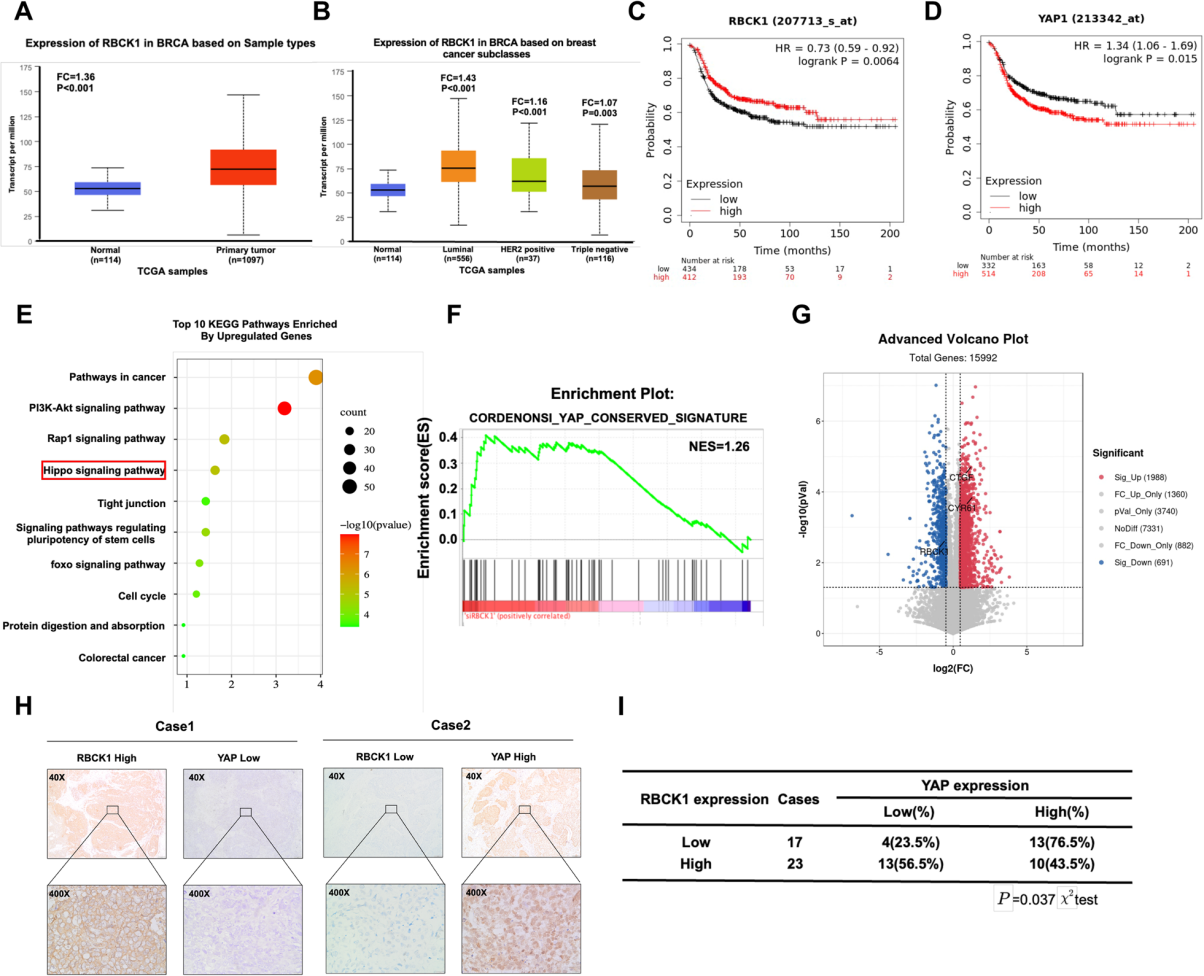
Clinical data analysis reveals the correlation between RBCK1 and Hippo signaling in TNBC **A** Compared with breast tissue in TCGA database, RBCK1 mRNA levels in breast cancer data samples increased.. (https://tcga-data.nci.nih.gov). **B** Compared with normal breast tissue in TCGA database, RBCK1 mRNA levels in all subtypes of breast cancer are increased. (https://tcga-data.nci.nih.gov). **C** Kaplan–Meier map of progression free survival demonstrates that RBCK1 is associated with good prognosis in triple negative breast cancer patient. (http://kmplot.com/analysis/). **D** Kaplan–Meier map of progression free survival demonstrates that YAP is associated with poor prognosis in triple negative breast cancer patients. **E** Gene ontology analysis of the RNA sequencing data shows that RBCK1 depletion in TNBC cells activates the Hippo signaling pathway. MDA-MB-231 cells were transfected with siRBCK1 or siControl. After 48 h, the total mRNA was extracted for RNA sequencing analysis. The siControl and siRBCK1 groups were analyzed in three times. **F** Gene set enrichment analysis (GSEA) analysis showed that the gene sets of CORDENONSI YAP CONSERVED SIGNATURE was enriched in the RBCK1-low group. **G** The volcanic map analysis showed that CTGF and CYR61 genes downstream of YAP were significantly up-regulated in the RBCK1-low group. **H** Cases of tumor showed that RBCK1 protein is negatively correlated with YAP protein in IHC. **I** Statistical analysis of RBCK1 correlation between RBCK1 and YAP in 40 TNBC tumor samples (Additional file 2)

We further analyzed the expression of RBCK1 in human sample to confirm the relationship between RBCK1 and TNBC. TCGA database showed that RBCK1 was moderately elevated in human breast cancers (Fold change = 1.36), while RBCK1 was also elevated all different subtypes compared with normal breast tissue (Luminal type, FC = 1.43; HER2 positive, FC = 1.16; TNBC, FC = 1.07) (Fig. 3A,B). In the prognosis analysis from KMPLOT database (https://kmplot.com), we found RBCK1 related to longer relapse-free survival in TNBC patients, which was opposite to previous studies regarding RBCK1 function in luminal types of breast cancers (Fig. 3C). Besides, we also observed that YAP expression related to shorter relapse-free survival in TNBC patients, which showed a reversed trend with RBCK1 prognosis (Fig. 3D). We used an unbiased manner to explore the function of RBCK1 in TNBC cells, then we use genome-wide expression analysis by depleting RBCK1 in MDA-MB-231 cells. KEGG pathway analysis suggested that RBCK1 deletion influenced a variety of cancer-related pathways, including Hippo signal (Fig. 3E). GSEA analysis confirmed that RBCK1 deletion evidently activated the YAP conserved gene signature (Fig. 3F). The volcano map analysis confirmed that RBCK1 silencing remarkably promoted the expression of Hippo classical target genes, containing CTGF and CYR61 (Fig. 3G). To verify this relationship, forty TNBC samples were gathered for immunohistochemical analysis. After processing, IHC data confirmed that YAP was located in cytoplasm and nucleus, while RBCK1 was mostly located in cytoplasm. Statistical analysis confirmed that the expression of RBCK1 was negatively relevant to the level of YAP protein (Fig. 3H,I) (Additional file 2).

### RBCK1 inhibits YAP protein level and Hippo target gene expression in TNBC

**Fig. 4:**
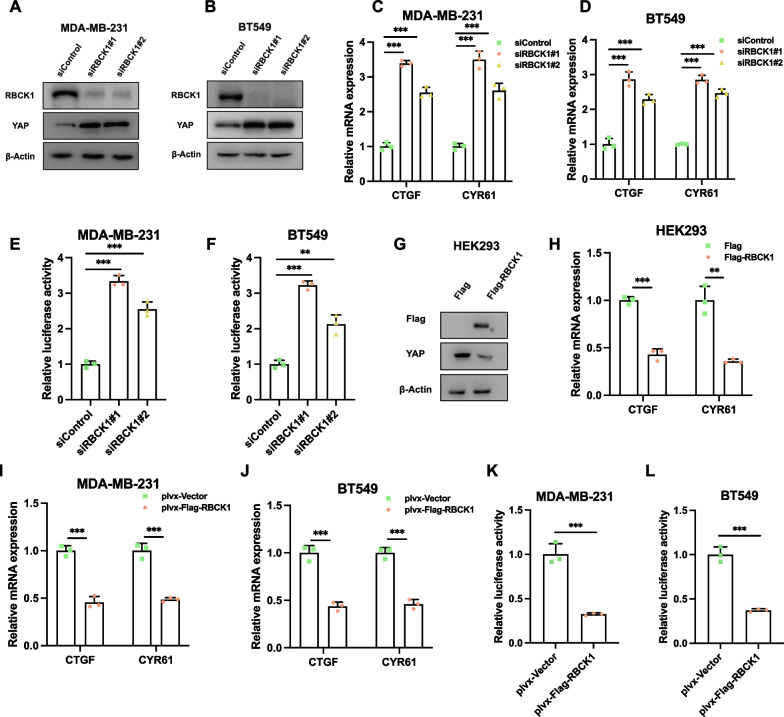
RBCK1 inhibits YAP protein level and Hippo target gene expression in TNBC **A**–**B** RBCK1 depletion increases the level of YAP protein in TNBC cells. MDA-MB-231 and BT549 cells were transfected with siControl or siRBCK1. 48 h later, the cells were collected for western blot analysis. **C**–**D** RBCK1 deletion increases expression of YAP target gene in TNBC cells. MDA-MB-231 and BT549 cells were transfected with siControl or siRBCK1. 48 h later, total RNA was collected for gene expression analysis. 36B4 was used as internal control. Each group was analyzed three times. *P < 0.05; **P < 0.01; ***P < 0.001 for target gene expression comparison. **E**–**F** RBCK1 deletion increased the activity of TEAD luciferase in TNBC cells. MDA-MB-231 and BT549 cells were transfected with siControl, siRBCK1,YAP luciferase reporter plasmid and Renilla plasmid. After 24 h, the cells were harvested for the detection of luciferase activity. Each group was analyzed three times. *P < 0.05; **P < 0.01; ***P < 0.001 for gene expression comparisons. **G** Overexpression of RBCK1 decreased the levels of YAP protein in HEK293 cells. HEK293 cells were transfected with Flag-RBCK1 or Flag-tag plasmids. 48 h later, the cells were collected for western blot analysis. **H** Overexpression of RBCK1 reduced the expression of YAP target gene in HEK293cells. HEK293 cells were transfected with Flag-RBCK1 or Flag-tag plasmids. After 48 h, total RNA was extracted for the gene expression analysis. 36B4 was used as internal control. Each group was analyzed three times. **I**–**J** Overexpression of RBCK1 reduced the expression of YAP target gene. MDA-MB-231 and BT549 cells with stable expression of RBCK1 or empty vector, total RNA was extracted for the gene expression analysis. 36B4 was used as internal control. Each group was analyzed three times. **K**–**L** Overexpression of RBCK1 reduced TEAD Luciferase activity in TNBC cells. MDA-MB-231 and BT549 cells with stable expression of RBCK1 or empty vector. After 24 h, the cells were transfected with the YAP luciferase reporter plasmid and Renilla plasmid. After 24 h, cells were harvested for the detection of luciferase activity. Each group was analyzed three times. *P < 0.05; **P < 0.01; ***P < 0.001 for gene expression comparisons

We further utilized two independent siRNAs for RBCK1 to avoid off-target effects. RBCK1 depletion could significantly increase YAP protein level in MDA-MB-231 and BT549 cells (Fig. 4A,B). Besides, we further detected the expression of Hippo target gene in TNBC cells. We found that RBCK1 deletion in MDA-MB-231 and BT549 cells boost the expression of Hippo target genes, containing CTGF and CYR61 (Fig. 4C,D). We further assessed the role of RBCK1 in Hippo signaling. Luciferase report analysis confirmed that the activity of TEAD response elements in MDA-MB-231 and BT549 cells were improved by RBCK1 silencing (Fig. 4E,F). In HEK293 cells, the protein levels of YAP and the expression of Hippo target gene, containing CTGF and CYR61, were decreased because of the expression of RBCk1 (Fig. 4G,H). The stable RBCK1 overexpression showed decrease Hippo target gene expression in MDA-MB-231 and BT549 cells (CTGF and CYR61) (Fig. 4I,J). The Luciferase reporter assay showed that RBCK1 overexpression in MDA-MB-231 and BT549 cells decreased TEAD response element activity (Fig. 4K,L).

### RBCK1 inhibits TNBC cancer progression via Hippo/YAP axis

**Fig. 5:**
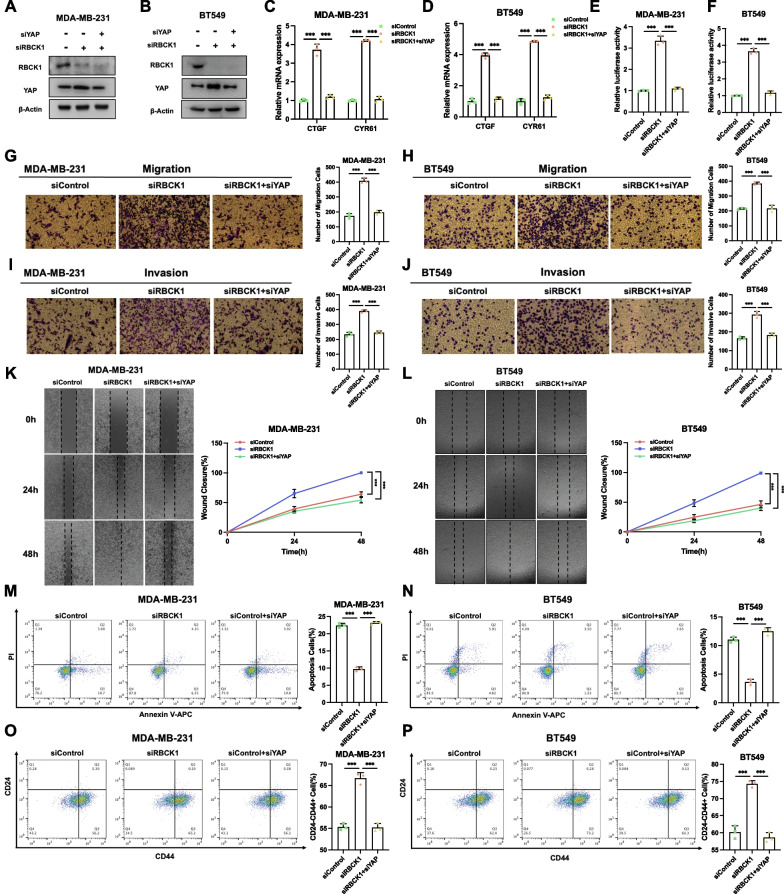
RBCK1 inhibits TNBC cancer progression via Hippo/YAP axis **A**–**B** RBCK1 deletion boosted the level of YAP protein, and the function can be saved after YAP deletion in TNBC cell. MDA-MB-231 and BT459 cells were transfected with siControl, siRBCK1 or siRBCK1 + siYAP. 48 h later,the cells were collected for western blot analysis. **C**–**D** RBCK1 deletion increased the expression of Hippo target gene, which can be reversed after YAP knocking-down in TNBC cell. MDA-MB-231 and BT459 cells were transfected with siControl, siRBCK1 or siRBCK1 + siYAP. 48 h later, total RNA was collected for gene expression analysis. 36B4 was used as internal control. Each group was analyzed three times. **E**–**F** RBCK1 deletion increased the activity of TEAD luciferase, which can be reversed after YAP knocking-down in TNBC cell. MDA-MB-231 and BT459 cells were transfected with siControl, siRBCK1 or siRBCK1 + siYAP., YAP luciferase reporter plasmid and Renilla plasmid. 24 h later, the cells were collected for the detection of luciferase activity. Each group was analyzed three times. *P < 0.05; **P < 0.01; ***P < 0.001 for gene expression comparisons. **G–H** RBCK1 depletion promotes the migration of cell, which can be reversed after YAP knocking-down in TNBC cell. MDA-MB-231 and BT459 cells were transfected with siControl, siRBCK1 or siRBCK1 + siYAP. We used the trans-well assay analysis to examine the invasion capability of TNBC cell. Calculate the number of cells and the number data showed as ± SD. *P < 0.05; **P < 0.01; ***P < 0.001 for cell comparisons. **I**–**J** RBCK1 depletion promotes the invasion of cell, which can be reversed after YAP knocking-down in TNBC cell. MDA-MB-231 and BT459 cells were transfected with siControl, siRBCK1 or siRBCK1 + siYAP. We used the trans-well assay analysis to examine the invasion capability of TNBC cell. Calculate the number of cells and the number data showed as ± SD. *P < 0.05; **P < 0.01; ***P < 0.001 for cell comparisons. **K**–**L** Wound-healing experiment confirmed that RBCK1 depletion increased the migration capacity of TNBC cells, the function could be saved after YAP deletion in TNBC cell. MDA-MB-231 and BT459 cells were transfected with siControl, siRBCK1 or siRBCK1 + siYAP. Wound closure was quantified at a specified point in time. Data are showed as ± SD. *P < 0.05; **P < 0.01; ***P < 0.001 for cell comparisons. **M**–**N** RBCK1 depletion inhibited apoptosis, which can be reversed after YAP knocking-down in TNBC cell. MDA-MB-231 and BT459 cells were transfected with siControl, siRBCK1 or siRBCK1 + siYAP. 24 h later, the cells were stained with PI and Annexin V. Then, FACS analysis was performed on the cells to determine the proportion of apoptotic cells. Each group was analyzed three times. **O**–**P** RBCK1 depletion increased the expression of CD24-CD44 + , in which can be reversed after YAP knocking-down in TNBC cell. MDA-MB-231 and BT459 cells were transfected with siControl, siRBCK1 or siRBCK1 + siYAP. After 24 h, cells were stained with anti-CD44-FITC and anti-CD24-PE. Then, FACS analysis was performed on the cells to determine the proportion of CD24-CD44 + cells. Each group was analyzed in three times

To study the logical relationship between TNBC cancer phenotype and Hippo/YAP signal in RBCK1 function, we conducted several rescue assays. RBCK1 deletion can increase the protein level of YAP, then YAP deletion in MDA-MB-231 and bt549 cells can save the protein level of YAP (Fig. 5A,B). QPCR experiment analysis confirmed that RBCK1 deletion can increase the expression of Hippo target genes containing CTGF and CYR61 and the function could be saved by YAP deletion in MDA-MB-231 and BT549 cells (Fig. 5C,D). Luciferase report analysis confirmed that the activity of TEAD response elements in MDA-MB-231 and BT549 cells were improved by RBCK1 silencing, the function can be saved by YAP deletion (Fig. 5E,F). In trans-well assays with permeable filter and basement membrane, TNBC cell invasion and migration could be boosted by RBCK1 deletion, and further YAP silencing could at least partially alleviate this effect (Fig. 5G–J). Wound healing experiment confirmed that RBCK1 deletion in MDA-MB-231 and BT549 cells could enhance the rate of cell migration, while YAP silencing could further save this function (Fig. 5K,L). The apoptosis experiment confirmed that the decreased cell death by RBCK1 depletion could be saved in MDA-MB-231 and BT549 cells by further YAP deletion (Fig. 5M,N). The increased stem cell proportion caused by RBCK1 depletion could be also rescued by further YAP depletion in MDA-MB-231 and BT549 cells (Fig. 5O,P). These data confirmed that Hippo signaling pathway could be regulated by RBCK1 in TNBC cells through YAP protein.

### RBCK1 relevant to YAP and regulates the stability YAP in TNBC cells

**Fig. 6:**
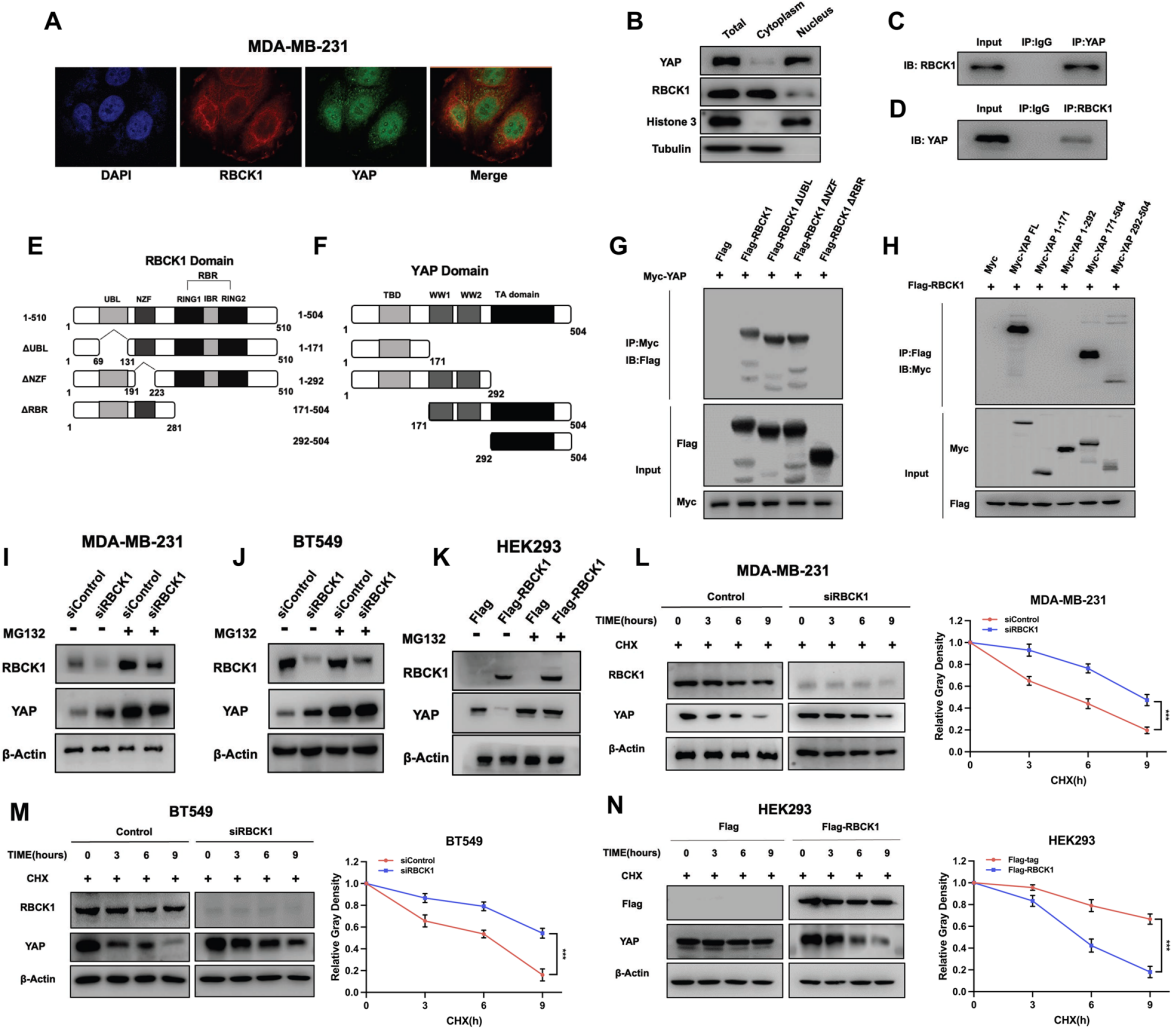
RBCK1 associates with YAP and modulates YAP stability in TNBC cells **A** The intracellular localization of RBCK1 and YAP was analyzed by immunofluorescence. MDA-MB-231 cells were cultured in normal medium before fixation. Intracellular localization of RBCK1 (red) and YAP (green) is shown. Nuclei (blue) were stained with 4’,6-diamidino-2-phenylindole (DAPI). **B** RBCK1 is mainly localized in the cytoplasm. YAP is mainly localized in the nuclear. We used a subcellular protein fractionation kit (Thermo Scientific, 78,840) for cytoplasm and nuclear separation experiment. **C**–**D** Co-IP experiment revealed the association between endogenous RBCK1 and YAP protein in MDA-MB-231 cells. MDA-MB-231 cells were collected with RIPA lysis buffer. CO-IP was performed using an antibody as indicated **E**–**F** RBCK1 full-length and deletion mutants are used in the study (Full-length, ΔUBL, ΔNZF, ΔRBR domains). YAP full-length and deletion mutants are used (Full length, ΔTA, ΔWW + ΔTA, ΔTBD, ΔTBD + ΔWW). **G** RBCK1 needs RBR domain to interact with YAP. HEK293 cells were transfected with 2 µg Myc-YAP and Flag-RBCK1 full-length or mutants (ΔUBL, ΔNZF, ΔRBR domains). 24 h later, the cells were collected with NP-40 lysis buffer. CO-IP was performed using an anti-Myc antibody. The interacting RBCK1 domains were detected by an anti-Flag antibody. **H** YAP needs TA domain to interact with RBCK1. HEK293 cells were transfected with 2 µg Flag-RBCK1 and Myc-YAP full-length or mutants (ΔTA, ΔWW + ΔTA, ΔTBD, ΔTBD + ΔWW). 24 h later, cells were harvested with NP-40 lysis buffer. The use of antibodies is shown in the figure. **I**–**J** RBCK1 did not further increase the level of YAP protein in TNBC cells in the presence of proteasome inhibitor MG132. MDA-MB-231 and BT549 cells were transfected with siControl or siRBCK1. 24 h later, the cells were treated with MG132 or vehicle for 6 h. The cell lysates were collected for western blot analysis. **K** The degradation effect of RBCK1 on YAP did not increase the levels of YAP protein any more in TNBC cell in the presence of the proteasome inhibitor MG132. HEK293 cells were transfected with 0.5 mg Flag-tag or Flag -RBCK1 plasmids. After 24 h, the cells were treated with 10 μM MG132/vehicle for 6 h. The cell lysates were prepared for western blot analysis. β-Actin was used as the internal reference. The results are representative of three independent experiments. **L**–**M** RBCK1 depletion increases the level of YAP half-life in TNBC cells. MDA-MB-231 and BT549 cells were transfected with siControl or siRBCK1. 24 h later, the cells were treated with 100 µM cycloheximide/vehicle for the specified time. The cell lysates were prepared for western blot analysis. The relative YAP density was measured by ImageJ software. β-Actin was used as the internal reference. The results are representative of three independent experiments. **N** Overexpression of RBCK1 reduced YAP half-life in HEK293 cells. HEK293 cells were transfected with 0.5 mg Flag-tag or Flag -RBCK1 plasmids. 24 h later, the cells were treated with 100 µM cycloheximide/vehicle for the specified times. The cell were collected for western blot analysis. The relative YAP density was measured by ImageJ software. β-Actin was used as the internal reference. The results are representative of three independent experiments

We investigated the location of RBCK1 and YAP in TNBC cells. Immuno-staining showed that rbck1 was mostly located in the cytoplasm, while YAP was mostly located in the nucleus (Fig. 6A). The nucleocytoplasmic separation test could further evidence this data analysis (Fig. 6B). RBCK1 could bind to YAP in TNBC cells, which could be confirmed by Endogenous immunoprecipitation (Fig. 6C,D). The RBCK1 protein is make up of UBL domain (Ubiquitin-like domain), NZF domain (Npl4 Zinc finger domain) and RBR (RING-in-between-RING) domain, while YAP protein is make up of TA domain (Transcription activation domain), WW domain (WW1 domain and WW2 domain) and TBD domain (TEAD binding domain) (Fig. 6E,F). We made these special plasmid structures to study the related domains through IP analysis. The immunoprecipitation confirms that the TA domain of YAP is required for the interaction with RBCK1, and the RBR domain of RBCK1 is required for its interaction with YAP (Fig. 6G,H). In MDA-MB-231 and BT549 cells, RBCK1 depletion could increase YAP protein level and MG132 treatment could save this effect (Fig. 6I,J). In addition, the overexpression of RBCK1 in HEK293 cells confirmed that RBCK1 could decrease the protein level of YAP and proteasome inhibitor MG132 could save this function (Fig. 6K). These western blot analyses suggest that YAP protein level could be regulated by RBCK1 because of proteasome degradation system. We further investigate the protein half-life level of YAP in TNBC cells, which suggested that RBCK1 depletion could improve YAP stability in MDA-MB-231 and BT549 cells (Fig. 6L,M). This was further confirmed in HEK293 cells via RBCK1 overexpression (Fig. 6N).

### RBCK1 functions as one ubiquitin ligase to promote YAP K48-linked poly-ubiquitination

**Fig. 7:**
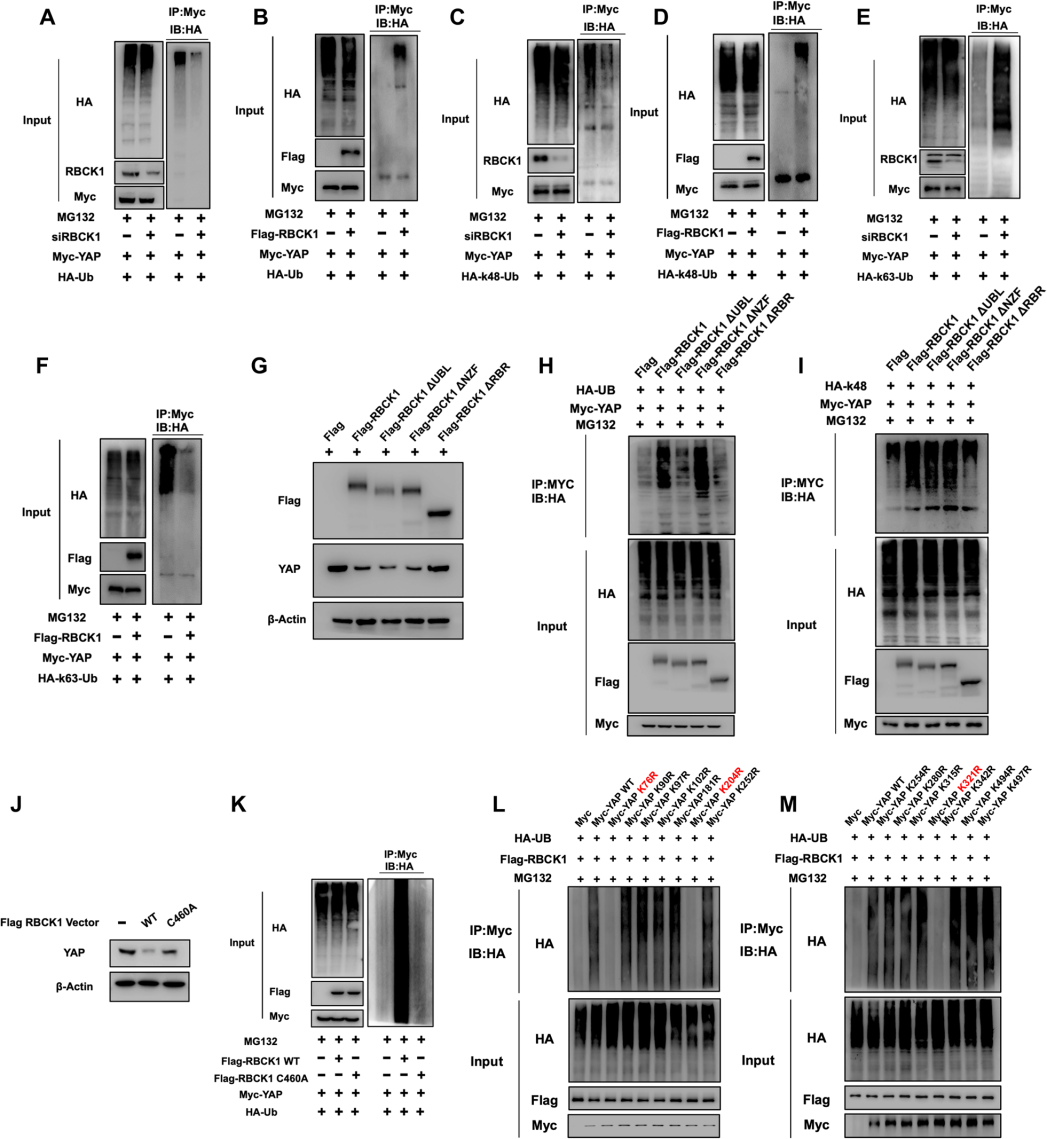
RBCK1 functions as one ubiquitin ligase to promote YAP K48-linked poly-ubiquitination** A** RBCK1 deletion decreases the total polyubiquitination of YAP. siControl or siRBCK1 were transfected into MDA-MB-231 cells. 24 h later, 1 mg HA-Ub plasmid and 2 µg YAP plasmid were transfected into cells. 24 h later, the cells were immunoprecipitated with an anti-HA antibody. **B** RBCK1 boosted total polyubiquitination of YAP. 2 µg of YAP plasmid, 0.5 µg of HA-Ub plasmid and 0.5 µg of Flag-tag or Flag -RBCK1 plasmids were transfected into HEK293T cells. After 24 h, the cells were immunoprecipitated with an anti-HA antibody. **C** RBCK1 deletion decreases the K48-linked polyubiquitination of YAP. siControl or siRBCK1 were transfected into MDA-MB-231 cells. 24 h later, 1 mg HA-K48 Ubi plasmid and 2 µg of YAP plasmid were transfected into cells. 24 h later, the cell were immunoprecipitated with an anti-HA antibody. **D** RBCK1 boosted the K48-linked polyubiquitination of YAP. 2 µg of YAP plasmid, 0.5 µg of HA-K48 Ubi plasmid and 0.5 µg of Flag-tag or Flag -RBCK1 plasmids were transfected into HEK293T cells. 24 h later, the cells were immunoprecipitated with an anti-HA antibody. **E** RBCK1 deletion boosted the K63-linked polyubiquitination of YAP. siControl or siRBCK1 were transfected into MDA-MB-231 cells. 24 h later, 1 mg HA-K63 Ubi plasmid and 2 µg of YAP plasmid were transfected into cells. 24 h later, the cell were immunoprecipitated with an anti-HA antibody. **F** RBCK1 decreases K63-linked polyubiquitination of YAP. 2 µg of YAP plasmid, 0.5 µg of HA-K63 Ubi plasmid and 0.5 µg of Flag-tag or Flag -RBCK1 plasmids were transfected into HEK293T cells. After 24 h, the cell extracts were immunoprecipitated with an anti-HA antibody. **G** RBCK1 inhibits YAP degradation by RBR domain. 2 µg Flag-RBCK1 full length or mutant plasmids (ΔUBL, ΔNZF, ΔRBR domains) were transfected into HEK293T cells. After 24 h, the cells were harvested directly and used for the western blot analysis. β-Actin was used as the internal reference. **H** The UBL domain or RBR domain of RBCK1 is important to improve total ubiquitination YAP. 2 µg, Myc-YAP, 1 µg HA-UB plasmid and 1 µg Flag-RBCK1 full length or mutants (ΔUBL, ΔNZF, ΔRBR domains) were transfected into HEK293 cells. The polyubiquitinated YAP was detected through the western blot analysis. **I** The RBR domain of RBCK1 is important to improve K48-linked ubiquitination of YAP. 2 µg Myc-YAP, 1 µg HA-K48 Ubi plasmid and 1 µg Flag-RBCK1 full length or mutants (ΔUBL, ΔNZF, ΔRBR domains) were transfected into HEK293 cells. The K48-specific polyubiquitinated YAP was detected through the western blot analysis. **J** The ability of RBCK1 to degrade YAP protein was impaired by the mutations of RBCK1 in MDA-MB-231 cells. **K** The mutations of RBCK1 that impaired ubiquitination of RBCK1 activity and the ability to increase the total polyubiquitination of YAP. HEK293 cells were transfected with 2 µg of YAP plasmid, 0.5 µg of HA-Ub plasmid and 0.5 µg of Flag-tag or Flag -RBCK1 plasmids or Flag-RBCK1 C460A. After 24 h, the cells were immunoprecipitated with an anti-HA antibody. The total polyubiquitinated YAP was detected via western blot analysis. **L**–**M** K76, K204, and K321 mutations (K76R, K204, and K321R) largely eliminated the ubiquitination effect of RBCK1 on YAP. The indicated vectors were transfected into HEK293 for ubiquitination analysis. Polyubiquitinated YAP was detected by Western blot analysis

Then we further examined the function of RBCK1, which is an E3 ubiquitin ligase, in YAP polyubiquitination. The polyubiquitination of YAP could be decreased by RBCK1 silencing in MDA-MB-231 cells through ubiquitination-based immunoprecipitation (Fig. 7A). Consistently, RBCK1 overexpression was shown to promote YAP overall poly-ubiquitination in HEK293 cells (Fig. 7B). In general, K48 linked ubiquitination is the classical manner for protein degradation mode, we studied the function of RBCK1 on K48 linked ubiquitination of YAP. In MDA-MB-231 cells, immunoprecipitation analysis showed that RBCK1 deletion could decrease K48 linked ubiquitination of YAP (Fig. 7C). In the HEK293 cells, we observed that RBCK1 overexpression could enhance K48-linked poly-ubiquitination (Fig. 7D). Interestingly, RBCK1 depletion could increase K63-linked ubiquitination in MDA-MB-231 cells, while RBCK1 overexpression could inhibit K63-linked ubiquitination in HEK293 cells (Fig. 7E). This might indicate RBCK1 could inhibit non-proteolytic ubiquitination, such as K63-linked ubiquitination (Fig. 7F). We further overexpressed RBCK1 full length or variants to see the effect on YAP protein level in HEK293 cells, which indicated that RBR domain was required for RBCK1 to inhibit YAP protein level (Fig. 7G). Further co-expression with RBCK1 full length or domain variants in HEK293 cells showed that RBR domain was required for RBCK1 to induce YAP total poly-ubiquitination and K48-linked poly-ubiquitination (Fig. 7H,I). Further on, we constructed the E3 ligase dominant negative form of RBCK1 (C460A) and carried out the ubiquitin-based immuno-precipitation assay. The data showed that the ubiquitin ligation activity of RBCK1 was required for inducing YAP poly-ubiquitination and degradation (Fig. 7J,K). We further studied the exact ubiquitin site of YAP through RBCK1. There are 13 lysine sites in YAP protein. Ubiquitination based IP confirmed that RBCK1 could promote the multi-ubiquitination of YAP at multiple sites (K204, K321 and K76) (Fig. 7L,M).

## Discussion

**Fig. 8:**
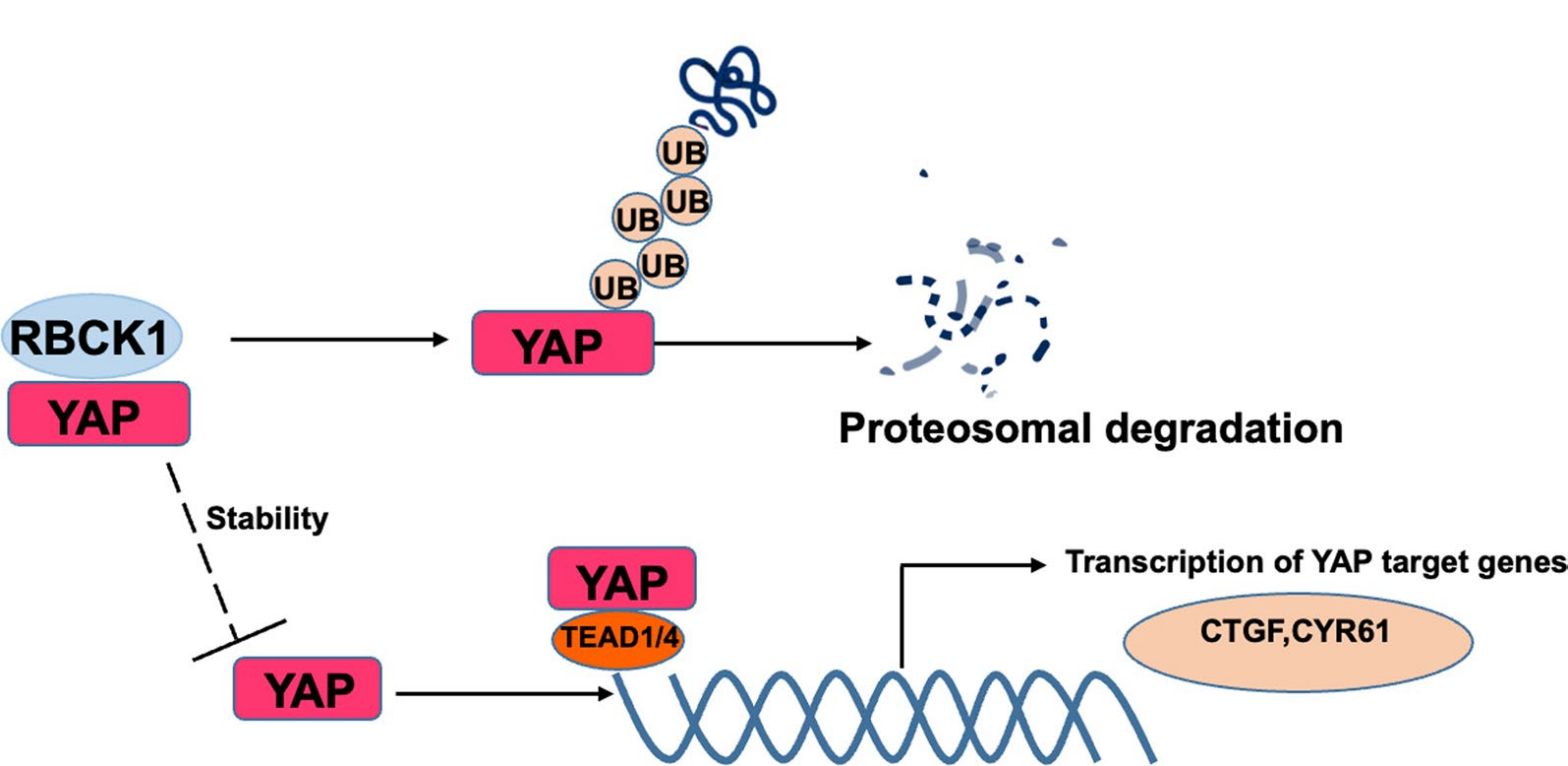
The hypothetical model for RBCK1 regulating Hippo/ YAP signaling in TNBC progression RBCK1 protein relevant to YAP and improve YAP degradation by leading YAP K48-linked polyubiquitination, which reduced the Hippo/YAP axis activation and invasion and migration of TNBC cells

In the current study, we report the E3 ubiquitin ligase RBCK1, which were regarded to facilitate estrogen signaling and luminal type breast cancer progression, exerts the opposite roles in TNBC subtypes. Although RBCK1 is modestly elevated in breast cancer and even elevated in TNBC, RBCK1 relates to good survival in TNBC and negatively correlates with YAP protein level in TNBC samples. RBCK1 inhibits TNBC cell progression and facilitates cell apoptosis via Hippo/YAP axis. The molecular and biological studies show that RBCK1 associates with YAP protein and facilitates YAP protein poly-ubiquitination and YAP degradation, which subsequently leads to the TNBC cell progression inhibition and apoptosis (Fig. 8). In summary, our study reveals the diversity of RBCK1 function in different subtypes of breast cancers.

Triple negative breast cancer is the most aggressive subtype of breast malignancies, which lacks of effective targets for therapy. Based on this, several studies investigated the potential biomarkers and therapeutic targets. For example, Farzaneh et al. showed that circ_0047303 was an important regulator for TNBC progression, which could be an important target for TNBC therapy. Besides, one genomic network analysis also revealed a regulatory loop among mir-135b, mir-29b and GATA3 in TNBC progression. Interestingly, several micro RNAs were also proved to associate with BRCA1 mutation in breast cancer. Despite the important roles of small RNAs, targeting these RNA is still pre-mature in clinics for TNBC treatments [33–35].

Hippo signaling abnormity was found to several human cancers, while the over-activation of Hippo/YAP axis is the major effector for carcinogenesis and cancer progression [18]. In TNBC, YAP gene amplification was commonly found, which might implicate the enhanced Hippo target gene expression [36]. Several studies showed TNBC cells showed enhanced cell proliferation, invasion, and stem cell-like phonotype, while YAP depletion or pharmaceutically targeting could cause TNBC cell growth inhibition or cell apoptosis in vitro and in vivo [37, 38]. The molecular studies revealed that YAP played critical role in TNBC progression, not only activation TEADs transcriptional function, but also crosstalk with several oncogenic transcriptional factors, including RUNX1, AP1 and GLIs [39, 40]. In addition, the activation of Hippo/YAP axis, which could also trans-activate PD-L1 expression and facilitate tumor immune evasion in TNBC, while the immune check-point inhibitors targeting PD1 could improve TNBC therapeutics [41, 42]. Based on the knowledge, we can propose that the over-activation of Hippo/YAP axis could be one of the major driver pathways for TNBC, while targeting YAP protein could be an effective strategy for TNBC treatment. It is still not clear why Hippo effector YAP protein is over-active, while the phosphorylation control of YAP activity by the serine/threonine kinases, such as MST1/2 and LATS1/2 remains intact. Recent studies revealed that other forms of post-translational modifications also play important role in YAP function, such as ubiquitination. For example, our previous study revealed that PARK2, which was decreased in human esophageal cancer, could induce YAP poly-ubiquitination and degradation. In our current study, we propose the novel E3 ubiquitin ligase RBCK1 in suppression Hippo/YAP signaling via promoting YAP K48-linked ubiquitination and degradation [26]. Our study gives a basis further understanding of the micro control of YAP protein in TNBC, but also proposed a promising target for rescuing Hippo signaling function in TBNC.

There are about 700 RING finger family proteins identified, most of which contain RING-In-Between-RING (RBR) domain [43]. Different with other types of E3 ubiquitin ligases, which mostly mediate the proteolytic poly-ubiquitination, recent studies revealed that several RING family ubiquitin ligases promoted atypical ubiquitination on their substrates. For example, RBCK could associate with RNF31 and SHARPIN, which form the linear ubiquitination assembly complex, mediates the linear ubiquitination of IKKr and promotes the activation of NFKB signaling [44]. Besides, RNF31 was also found to induce the mono-ubiquitination of ER alpha and breast cancer progression. However, it is still not totally clear for most of the RING family E3 ubiquitin ligase [45]. RBCK1 was initially identified from the yeast two-hybrid screen as a PKC interaction protein. Further structure analysis revealed that the RBR domain is the functional domain for E3 ubiquitin ligation [46]. Besides the function of RBCK1 in LUBAC complex and NFKB signaling, the function of RBCK1 in breast cancer were extensively studies in recent years. Nina et al. firstly reported that RBCK1 could act as one co-activator both transcriptional inducing ER alpha expression and trans-activation ER alpha function [30]. Further studies revealed that RBCK1 could enhance ER alpha signaling and facilitate tamoxifen resistance. Besides, there are also studies showing RBCK1 could inhibit P53 function and facilitate P53 degradation [47]. Based on these studies, we conclude RBCK1 was mostly regarded as an oncogene in cancers. However, there is no ER alpha expression and P53 mutant status in TNBC, the function of RBCK1 is largely unknown [29]. Although several studies implicated that RBCK1 could potentially been an oncogene, our understanding is that RBCK1 could act its roles in cancer type dependent manner. Our study confirmed that RBCK1 can decrease the progression of TNBC cells by inhibiting Hippo/Yap axis. This may be the first study to show the tumor suppressive effect of RBCK1 in cancer progression. These interesting findings boost the understanding of the regulation of Hippo/YAP signal transduction, but also reveal the "multifaceted" role of RBCK1 in different subtypes of breast cancer.

## Conclusions

Our study revealed an unexpected function of RBCK1 in TNBC progression. RBCK1 could associate with YAP protein and promote YAP protein K48-linked poly-ubiquitination and degradation, which subsequently inhibited YAP-driven signaling function and TNBC progression. Pharmaceutically activation RBCK1 function or inducing RBCK1 expression could be a promising strategy for TNBC treatments.

## Supplementary Information

**Additional file 1**. Cell STR test report.

**Additional file 2**. Clinicopathological Data for 40 TNBC.

**Additional file 3**. Digital raw data.

**Additional file 4**. Western Blot original image.

## Data Availability

Publicly available data is available in the GEO database (accession number: GSE195712).

## Abbreviations

TNBC: Triple negative breast cancer
RBCK1: RANBP2-type and C3HC4-type zinc finger-containing 1
RBR: RING-between-RING domain
NZF: Npl4 zinc finger domain
YAP: Yes-associated protein
TEAD: TEA domain transcriptional factor
RING: Really interesting new gene
LATS: Large tumor suppressor kinase

## Declarations

Digital raw data and Western Blot original image is provided in the Supplementary Information (Additional files 3, 4)
