# Supplementary material for: RBCK1 is an endogenous inhibitor for triple negative breast cancer via hippo/YAP axis
Source: Cell Commun Signal. 2022 Oct 24;20:164. doi: 10.1186/s12964-022-00963-8 (PMC9590148; doi:10.1186/s12964-022-00963-8)

www.microread.com

阅 微 基 因　　省 时 省 心

细胞 STR 检验报告

委托单位：新乡医学院检验学院

委托项目：细胞 STR 检验

委托日期：2017-11-06

细胞 STR 检验报告

委托方：新乡医学院检验学院

委托日期：2017 年 11 月 06 日

鉴定日期：2017 年 11 月 13 日

一、 检材：

2017 年 11 月 06 日收到委托人 1 株细胞悬液：

编号为“XB6569”，包装上标记为“BT549”。

要求鉴定该株细胞是否为单一来源细胞系, 是否存在交叉污染现象。

二、 检材处理和检验方法：

取适量检材用 Microread Genomic DNA Kit 提取 DNA，采用 Microreader^{TM}21 ID System 扩增 20 个 STR 位点和性别鉴定位点，使用 ABI 3730xl 型遗传分析仪进行 PCR 产物检测，使用 GeneMapper3.2 软件(Applied Biosystems)对检测结果进行分析，并与 ATCC 和 DSMZ 数据库进行比对。

三、 检验结果：

实验中阴性及阳性对照结果均正确。

该细胞株的 STR 位点和 Amelogenin 位点的基因分型结果见附表，分型图谱见附图。

Genetic Site	ATCC				检测样品				
(Locus)	数据库名称：BT549Breast AdenocarcinomaHuman				样品名称:BT549				
Amelogenin	X				X				
D5S818	11	12			11	12			
D13S317	11				11				
D7S820	8	9			8	9			
D16S539	11	12			11	12			
vWA	14	15			14	15			
TH01	6				6				
TPOX	9	12			9	12			
CSF1PO	10				10				

所有匹配峰的个数	14
ATCC 数据库所有峰的个数	14
匹配度	100%

四、 分析说明：

该细胞 DNA 扩增后图谱清晰，分型结果良好。

五、 检验结论：

BT549:①该株细胞 DNA 进行细胞 STR 分型结果显示，在各基因座均未出现三等位基因现象。细胞中没有发现人类细胞交叉污染。② BT549 细胞与 ATCC BT549 Breast AdenocarcinomaHuman 细胞的 STR 数据匹配率为 100%，推测此样品为 BT549 Breast AdenocarcinomaHuman。③该株细胞 DNA 分型在 DSMZ 细胞库中找到与其细胞分型 100%相匹配的细胞，该细胞名称为 BT549 等。

（此结果仅对本次检材负责）

操作人：王蕊　　　　审核人：吴炳君

北京阅微基因技术有限公司

备注：

1. 根据 ANSI 制定的国际标准，细胞系的匹配度≥80% 时，认为它们具有相关性，即衍生于共同的祖先细胞；匹配度在 55% 至 80% 之间，需要进一步验证相关性；小于 55%，表明两者不具有相关性。
2. 有效峰为真实的 PCR 条带;小峰和非特异性条带在计算中忽略不计。

本实验依照 Microreader™21 ID System STR 试剂盒提供的实验手段和分析方法进行检测，结果仅供参考。

Standards for Cell Line Authentications

To standardize STR analysis for human cell line authentication, the American Tissue Culture Collection (ATCC) Standards Development Organization Workgroup published ASN-0002-2011, which recommends the use of at least eight STR loci (TH01, TPOX, vWA, CSF1PO, D16S539, D7S820, D13S317 and D5S818) plus Amelogenin for gender identification for human cell line authentication.

附表：细胞 BT549 的 STR 位点和 Amelogenin 位点的基因分型结果

细胞 BT549（图片编号 XB6569）		
Marker	Allele 1	Allele 2
D19S433	13	14
D5S818	11	12
D21S11	30	30
D18S51	14	14
D6S1043	12	18
AMEL	X	X
D3S1358	16	16
D13S317	11	11
D7S820	8	9
D16S539	11	12
CSF1PO	10	10
Penta D	12	12
D2S441	10	14
vWA	14	15
D8S1179	10	14
TPOX	9	12
Penta E	7	12
TH01	6	6
D12S391	18	20
D2S1338	21	23
FGA	23	25

MDA-MB-231 细胞 STR 鉴定报告

一、 材料处理和检验方法

取适量 **MDA-MB-231** 细胞(1×10^6)使用 PureLink® Genomic DNA Mini Kit（美国 Life K182001）提前基因组 DNA，采用 PowerPlex®18D 系统（美国 Promega DC1802）试剂盒进行扩增，在 ABI3500 Genetic Analyzer（美国 Life3500）进行检测。

二、 检测结果

实验中阴性及阳性对照结果均正确。

MDA-MB-231 细胞株的 STR 位点和 Amelogenin 位点的基因分型结果见附表，分型图谱见附图。

三、 分析说明

MDA-MB-231 细胞株基因组 DNA 扩增后图谱清晰，分型结果良好。

四、 检验结论

1. **MDA-MB-231** 细胞株 DNA 进行细胞 STR 分型结果显示，细胞株中未发现人类细胞交叉污染。
2. 该细胞株 DNA 分型在 ATCC 细胞库中找到与其细胞分型 100%相匹配的细胞株，细胞株名称为 **MDA-MB-231**。

Procell
www.procell.com.cn

网站：www.procell.com.cn
电话：400-650-3656
邮箱：sales@procell.com.cn

附表 1：细胞株 **MDA-MB-231** 的 **STR** 位点和 **Amelogenin** 位点的基因分型结果

细胞 **MDA-MB-231**（图片编号为 PC03）		
Marker	***Allele 1***	***Allele 2***
D3S1358	16	16
**THO1**	7	9.3
D21S11	33.2	33.2
D18S51	11	16
Penta E	11	11
**D5S818**	12	12
**D13S317**	13	13
**D7S820**	8	9
**D16S539**	12	12
**CSF1PO**	12	13
Penta D	11	14
**AMEL**	X	X
**Vwa**	15	18
D8S1179	13	13
**TPOX**	8	9
FGA	22	23

附图 1：**ATCC** 官网 **MDA-MB-231** 细胞 **STR** 位点信息

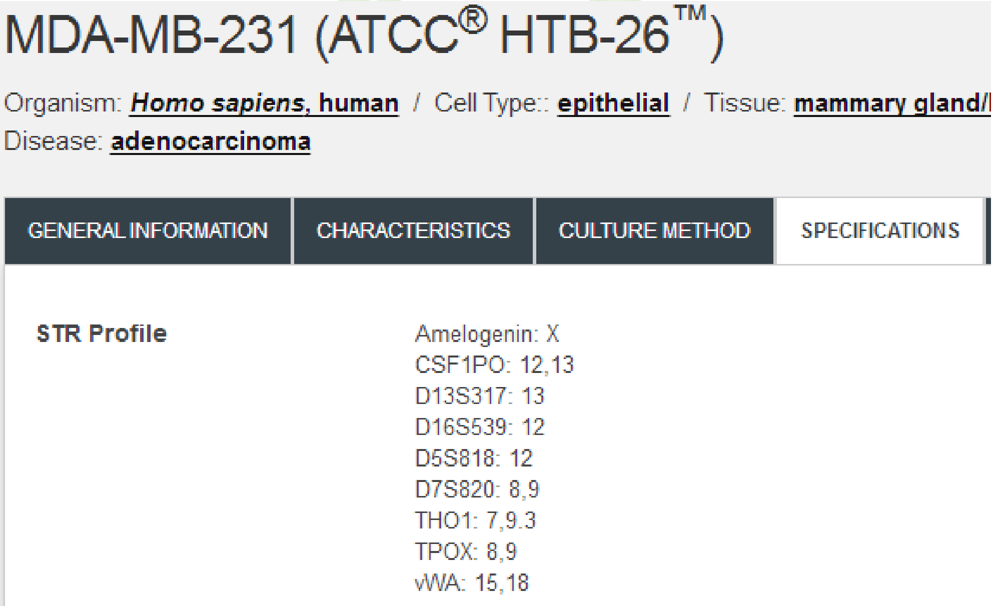
MDA-MB-231 (ATCC® HTB-26™)

Organism: **Homo sapiens, human** / Cell Type:: **epithelial** / Tissue: **mammary gland/**
Disease: **adenocarcinoma**

GENERAL INFORMATION | CHARACTERISTICS | CULTURE METHOD | SPECIFICATIONS

STR Profile

Amelogenin: X
CSF1PO: 12,13
D13S317: 13
D16S539: 12
D5S818: 12
D7S820: 8,9
THO1: 7,9.3
TPOX: 8,9
vWA: 15,18

附图 2：**MDA-MB-231** 细胞 **STR** 位点和 **Amelogenin** 位点的基因分型结果

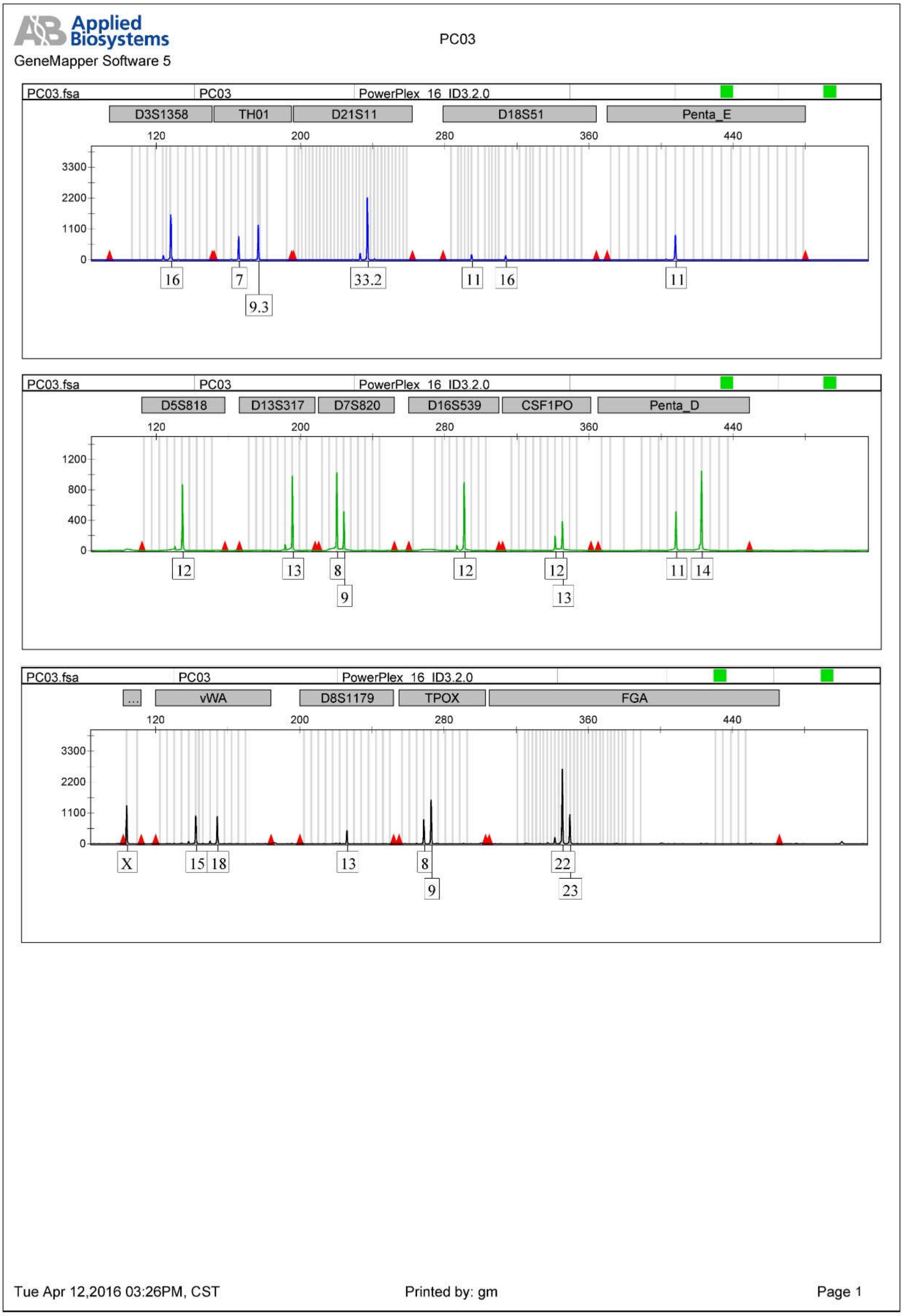

网站：www.procell.com.cn

电话：400-650-3656

邮箱：sales@procell.com.cn

www.microread.com

阅微基因 microread

阅 微 基 因　省 时 省 心

细胞 STR 检验报告

委托单位：新乡医学院检验学院

委托项目：细胞 STR 检验

委托日期：2017-11-06

细胞 STR 检验报告

委托方：新乡医学院检验学院

委托日期：2017 年 11 月 06 日

鉴定日期：2017 年 11 月 13 日

一、 检材：

2017 年 11 月 06 日收到委托人 1 株细胞悬液：

编号为“XB6570”，包装上标记为“HEK293”。

要求鉴定该株细胞是否为单一来源细胞系, 是否存在交叉污染现象。

二、 检材处理和检验方法：

取适量检材用 Microread Genomic DNA Kit 提取 DNA，采用 Microreader^{TM}21 ID System 扩增 20 个 STR 位点和性别鉴定位点，使用 ABI 3730xl 型遗传分析仪进行 PCR 产物检测，使用 GeneMapper3.2 软件(Applied Biosystems)对检测结果进行分析，并与 ATCC 和 DSMZ 数据库进行比对。

三、 检验结果：

实验中阴性及阳性对照结果均正确。

该细胞株的 STR 位点和 Amelogenin 位点的基因分型结果见附表，分型图谱见附图。

Genetic Site	ATCC				检测样品				
(Locus)	数据库名称: HEK-293.2susEmbryonic KidneyHuman				样品名称: HEK293				
Amelogenin	X				X				
D5S818	8				8				
D13S317	12	14			12	14			
D7S820	11	12			11				
D16S539	9	13			9	13			
vWA	16	19			16	19			
TH01	7	9.3			7	9.3			
TPOX	11				11				

CSF1PO	12				12				
所有匹配峰的个数									13
ATCC 数据库所有峰的个数									14
匹配度									93%

四、 分析说明：

该细胞 DNA 扩增后图谱清晰，分型结果良好。

五、 检验结论：

HEK293:①该株细胞 DNA 进行细胞 STR 分型结果显示，在各基因座均未出现三等位基因现象。细胞中没有发现人类细胞交叉污染。② HEK293 细胞与 ATCC 中 HEK-293.2susEmbryonic KidneyHuman 细胞的 STR 数据匹配率为 93%，推测此样品为 HEK-293.2susEmbryonic KidneyHuman 的衍生细胞。③该株细胞 DNA 分型在 DSMZ 细胞库中未找到与其细胞分型 100%相匹配的细胞。

（此结果仅对本次检材负责）

操作人：王蕊　　　　　　　　审核人：吴炳君

北京阅微基因技术有限公司

备注：

1. 根据 ANSI 制定的国际标准，细胞系的匹配度≥80% 时，认为它们具有相关性，即衍生于共同的祖先细胞；匹配度在 55% 至 80% 之间，需要进一步验证相关性；小于 55%，表明两者不具有相关性。
2. 有效峰为真实的 PCR 条带;小峰和非特异性条带在计算中忽略不计。

本实验依照 Microreader™21 ID System STR 试剂盒提供的实验手段和分析方法进行检测，结果仅供参考。

Standards for Cell Line Authentications

To standardize STR analysis for human cell line authentication, the American Tissue Culture Collection (ATCC) Standards Development Organization Workgroup published ASN-0002-2011, which recommends the use of at least eight STR loci (TH01, TPOX, vWA, CSF1PO, D16S539, D7S820, D13S317 and D5S818) plus Amelogenin for gender identification for human cell line authentication.

附表：细胞 HEK293 的 STR 位点和 Amelogenin 位点的基因分型结果

细胞 HEK293（图片编号 XB6570）		
Marker	Allele 1	Allele 2
D19S433	15	18
D5S818	8	8
D21S11	30.2	30.2
D18S51	17	17
D6S1043	11	11
AMEL	X	X
D3S1358	15	17
D13S317	12	14
D7S820	11	11
D16S539	9	13
CSF1PO	12	12
Penta D	9	9
D2S441	11	15
vWA	16	19
D8S1179	12	14
TPOX	11	11
Penta E	7	15
TH01	7	9.3
D12S391	19	21
D2S1338	19	19
FGA	23	23

附图：细胞 HEK293 的 STR 分型图谱

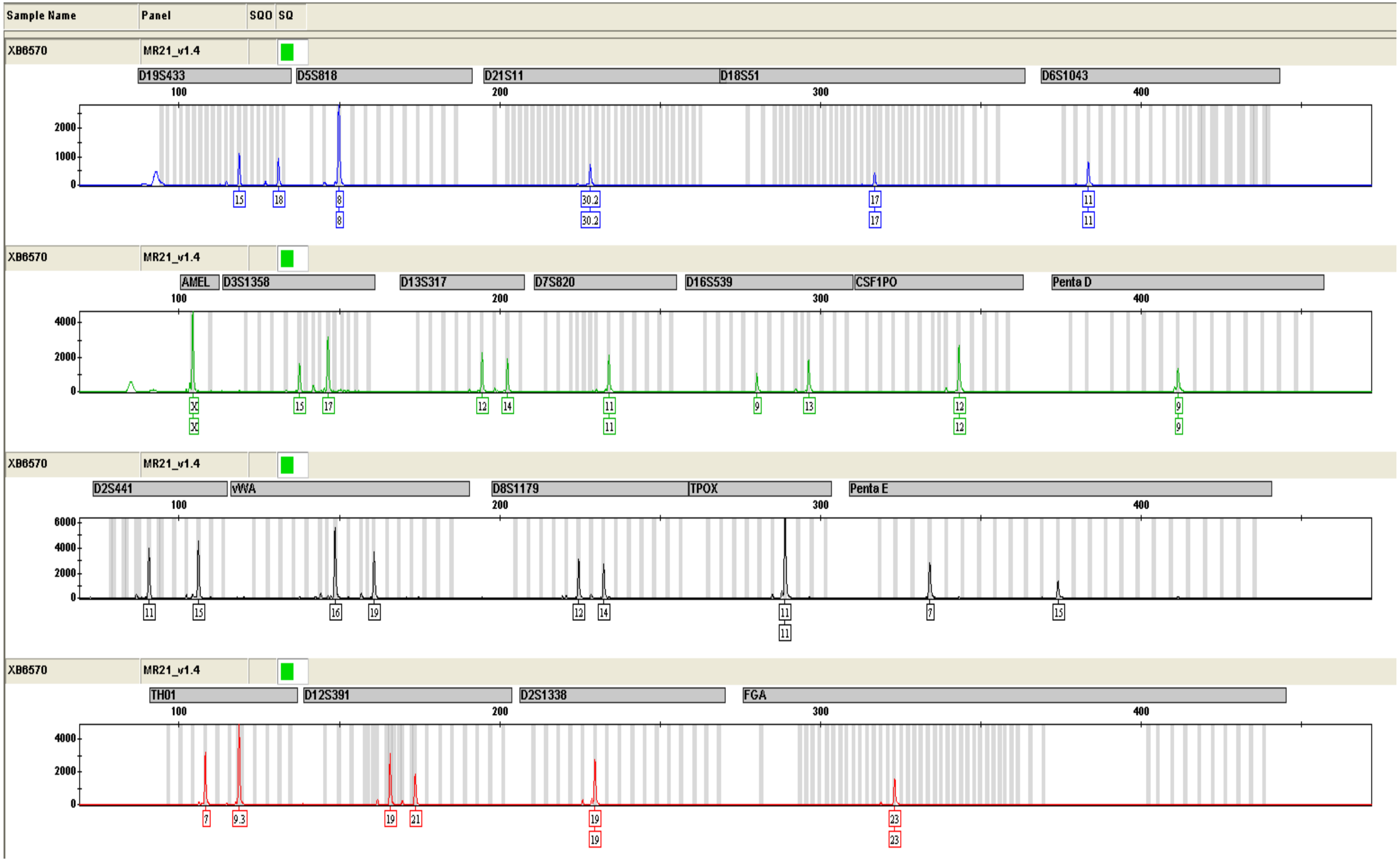

Supplement: Supplementary file 2 — Additional file 1. Cell STR test report. [file 12964_2022_963_MOESM2_ESM.pdf]
