# Supplementary material for: RBCK1 is an endogenous inhibitor for triple negative breast cancer via hippo/YAP axis
Source: Cell Commun Signal. 2022 Oct 24;20:164. doi: 10.1186/s12964-022-00963-8 (PMC9590148; doi:10.1186/s12964-022-00963-8)
Supplement: Supplementary file 5 — Additional file 4. Western Blot original image. [file 12964_2022_963_MOESM5_ESM.pdf]

Figure1

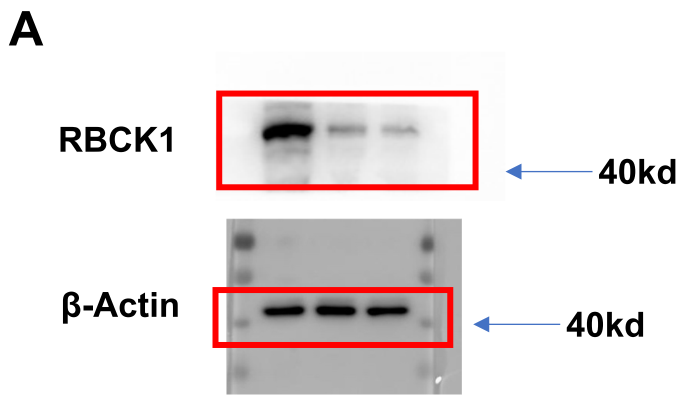

Figure 2

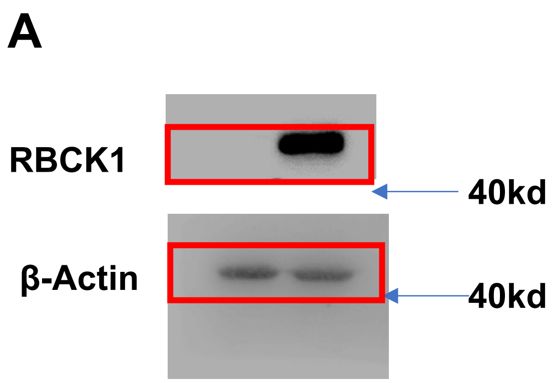

Figure 4

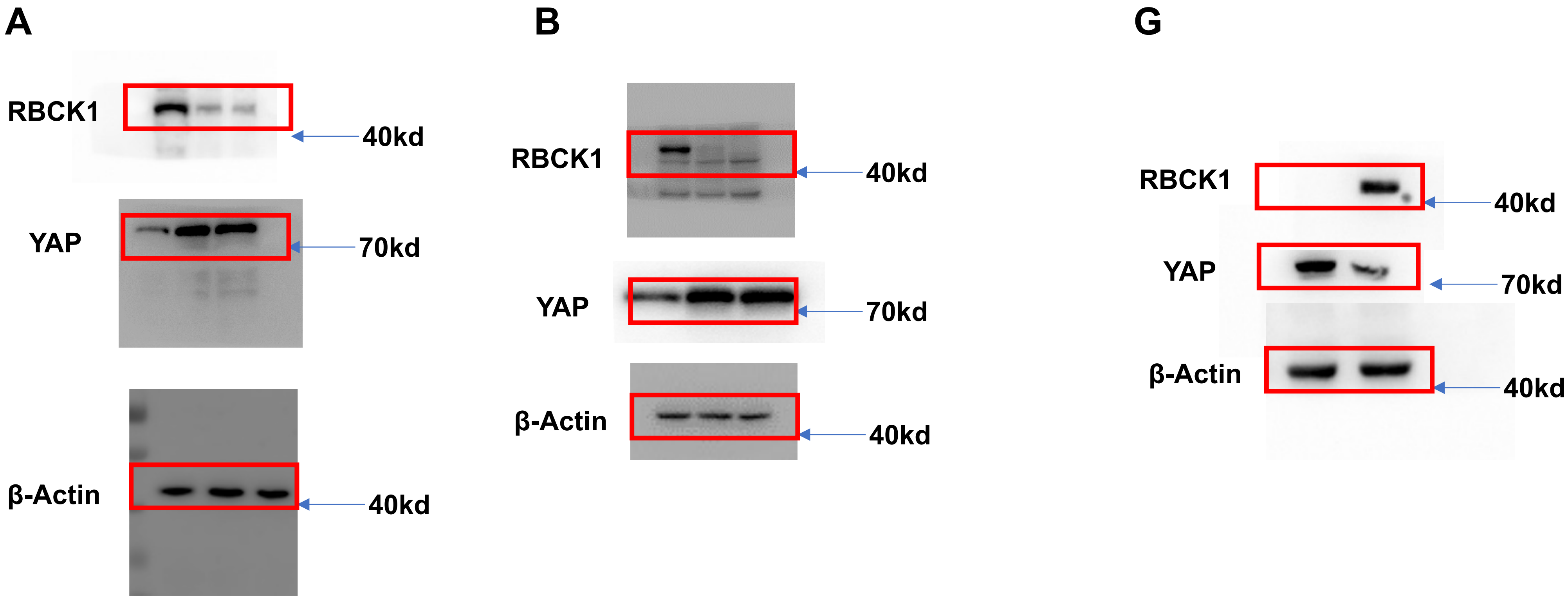
A
RBCK1
40kd
YAP
70kd
β-Actin
40kd
B
RBCK1
40kd
YAP
70kd
β-Actin
40kd
G
RBCK1
40kd
YAP
70kd
β-Actin
40kd

Figure 5

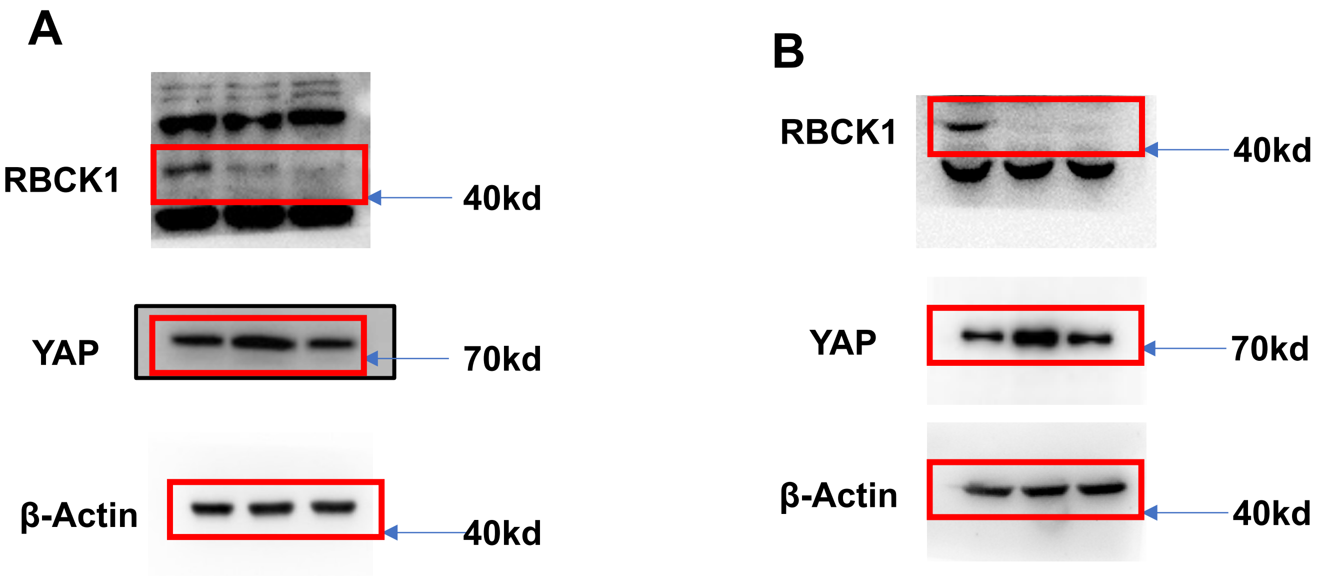
A
RBCK1
40kd
YAP
70kd
β-Actin
40kd
B
RBCK1
40kd
YAP
70kd
β-Actin
40kd

Figure 6

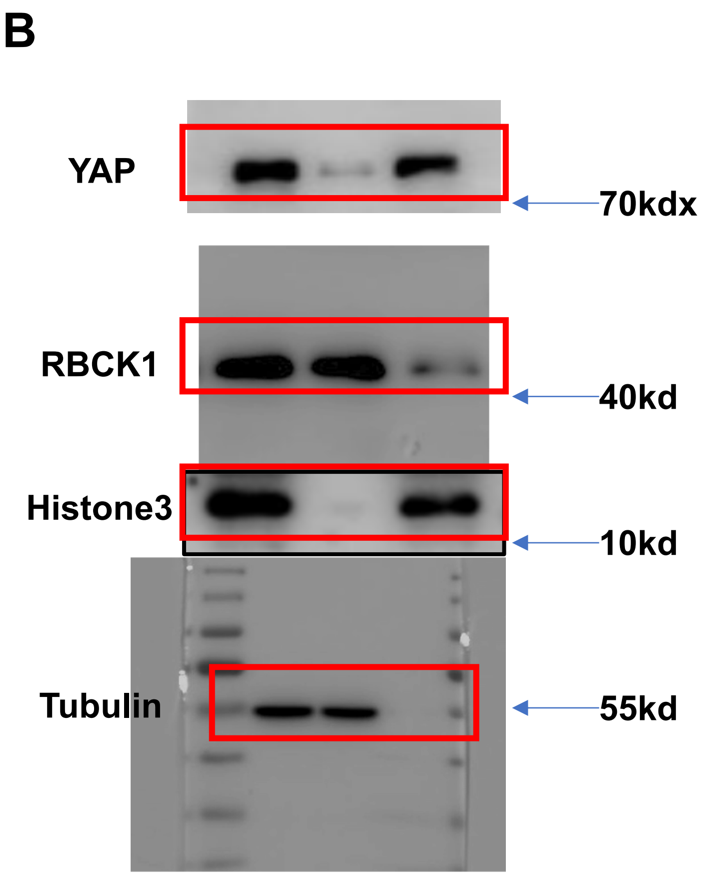
B
YAP
70kdx
RBCK1
40kd
Histone3
10kd
Tubulin
55kd

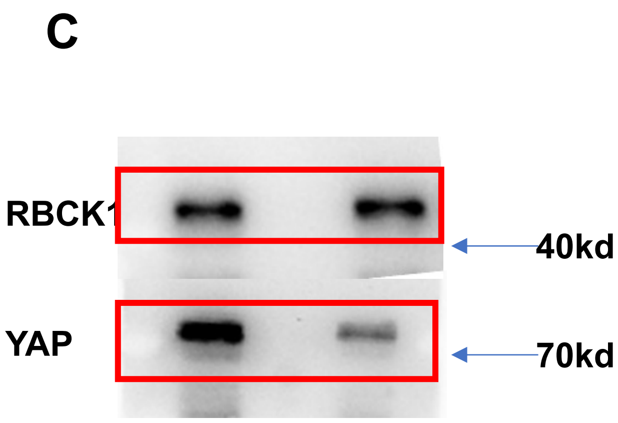
C
RBCK1
40kd
YAP
70kd

Figure 6

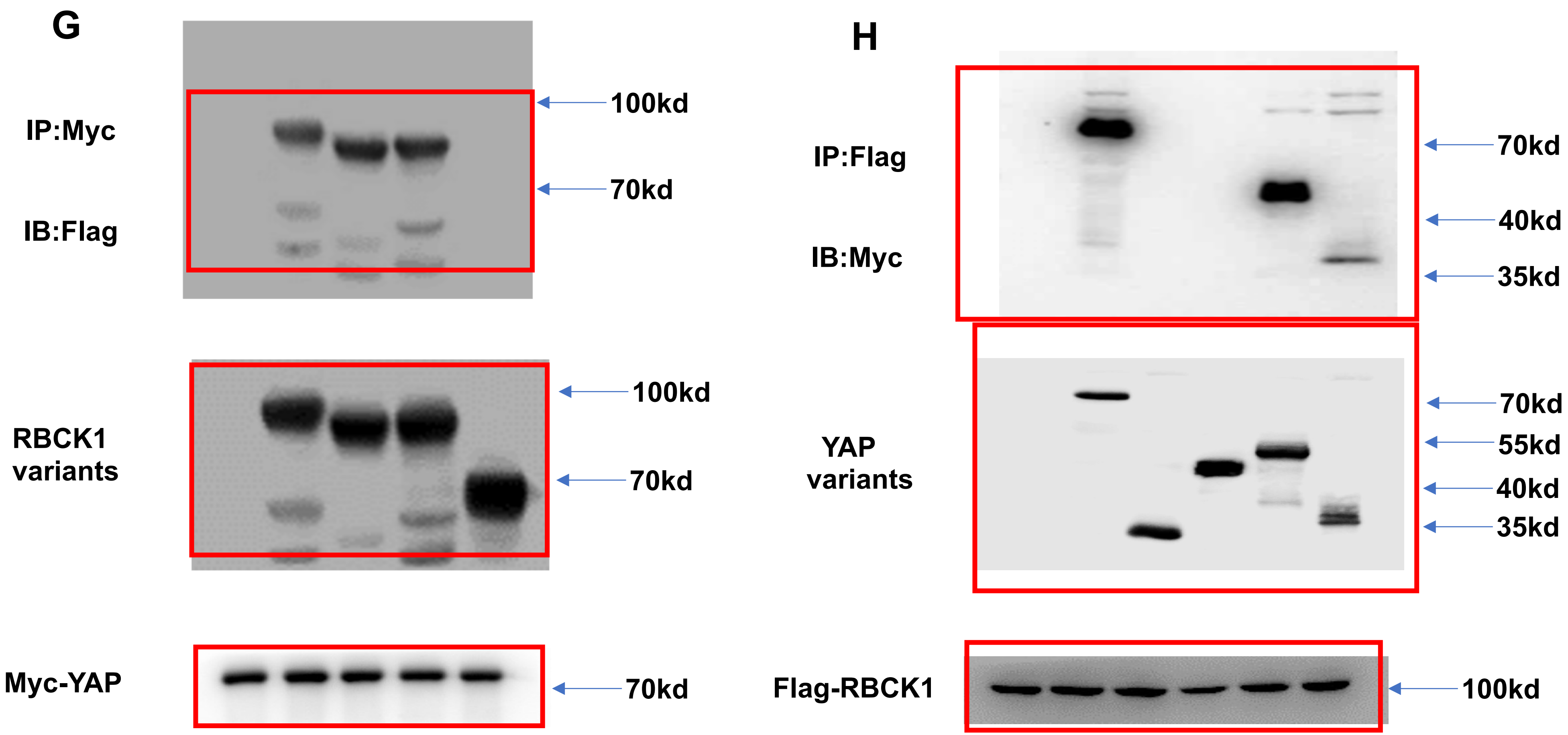
G
IP:Myc
IB:Flag
100kd
70kd
RBCK1
variants
100kd
70kd
Myc-YAP
70kd
H
IP:Flag
IB:Myc
70kd
40kd
35kd
YAP
variants
70kd
55kd
40kd
35kd
Flag-RBCK1
100kd

Figure 6

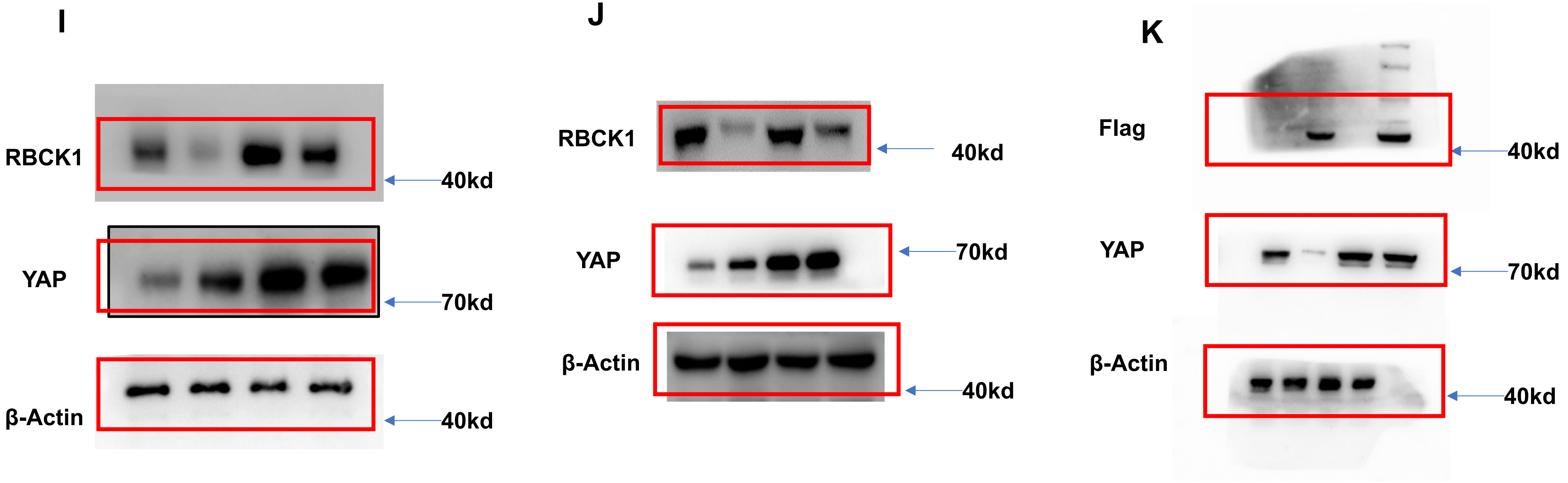

I
RBCK1
40kd
YAP
70kd
β-Actin
40kd
J
RBCK1
40kd
YAP
70kd
β-Actin
40kd
K
Flag
40kd
YAP
70kd
β-Actin
40kd

Figure 6

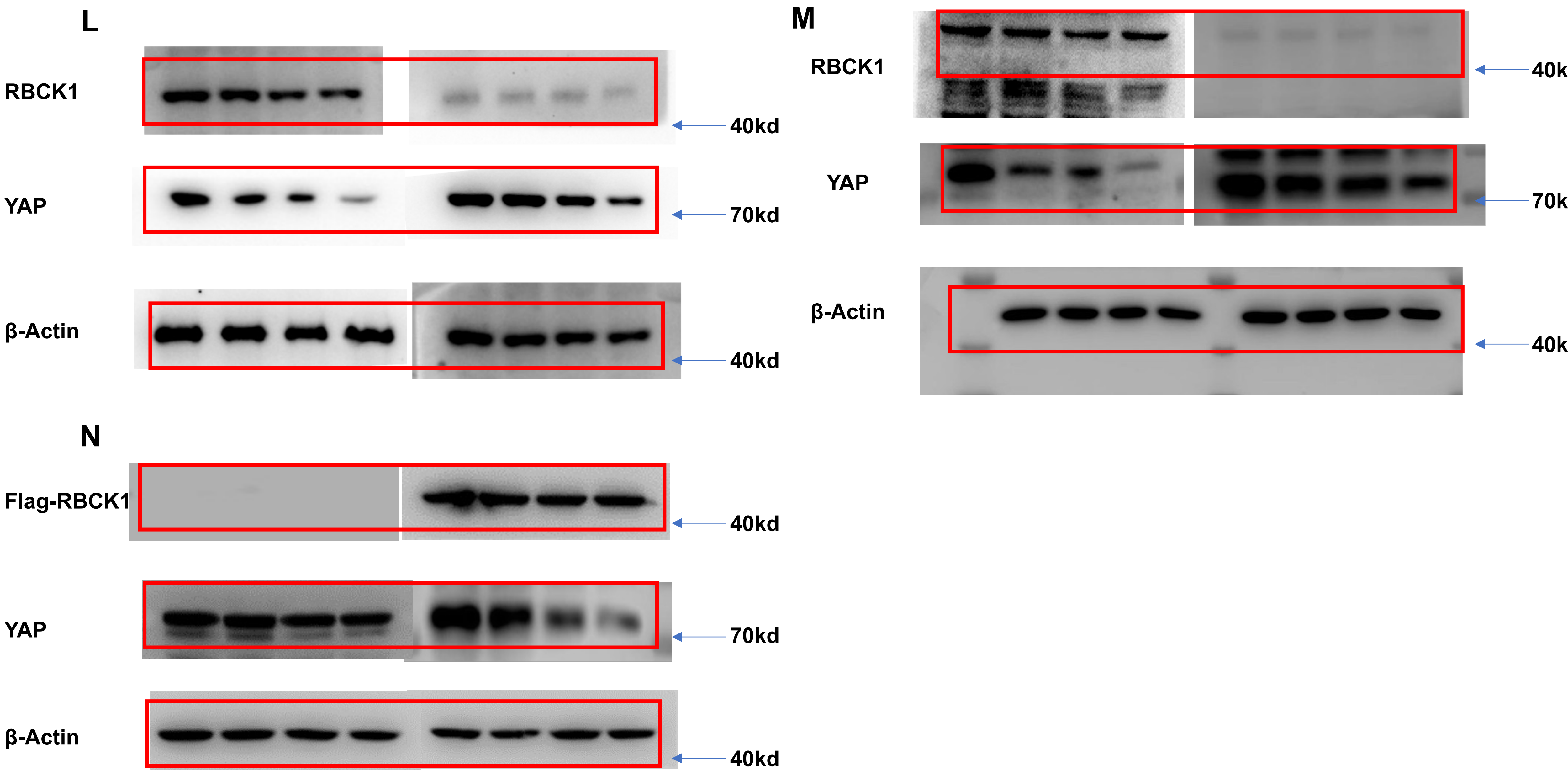
L
RBCK1
YAP
β-Actin
40kd
70kd
40kd
M
RBCK1
YAP
β-Actin
40k
70k
40k
N
Flag-RBCK1
YAP
β-Actin
40kd
70kd
40kd

Figure 7

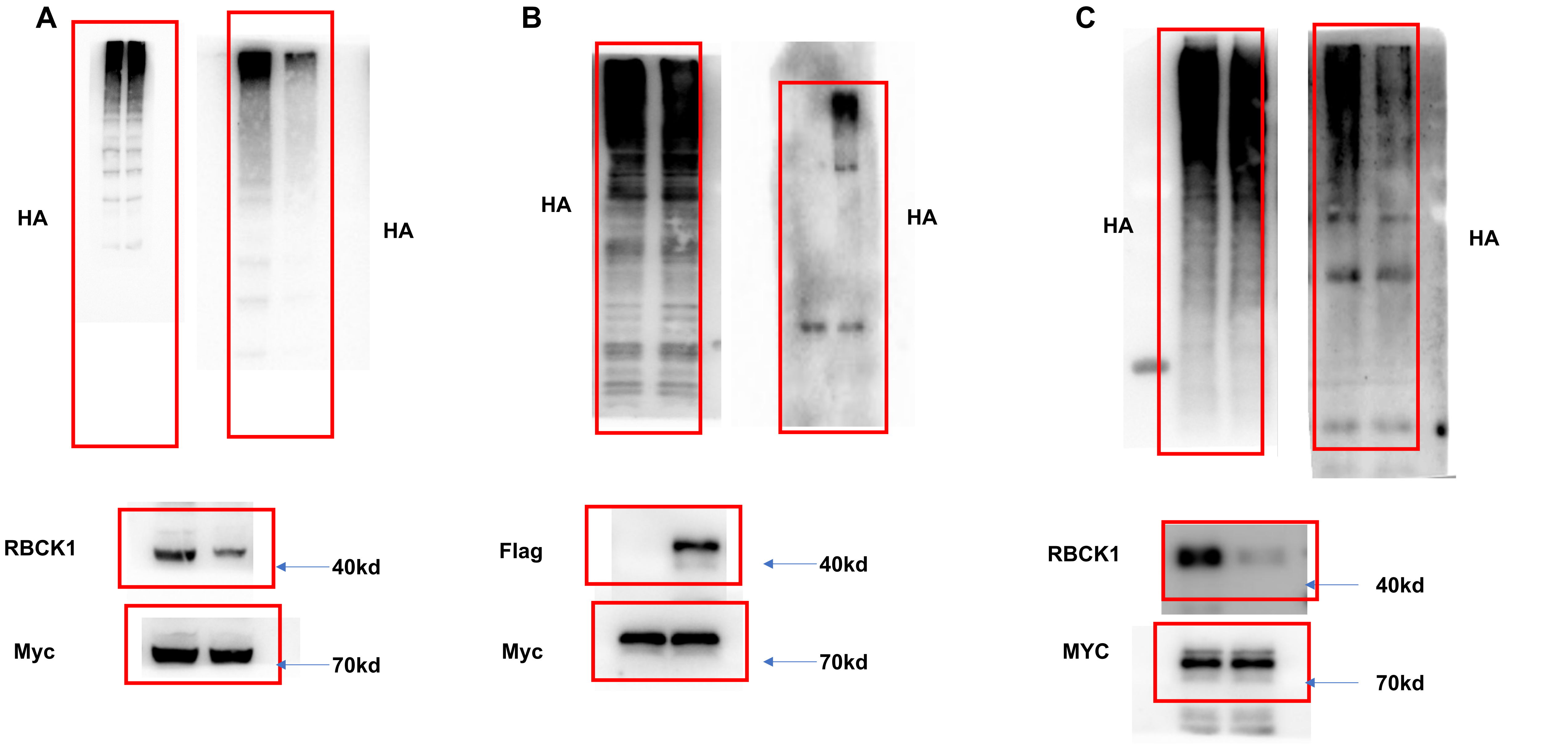

A
HA
HA
RBCK1
40kd
Myc
70kd
B
HA
HA
Flag
40kd
Myc
70kd
C
HA
HA
RBCK1
40kd
MYC
70kd

Figure 7

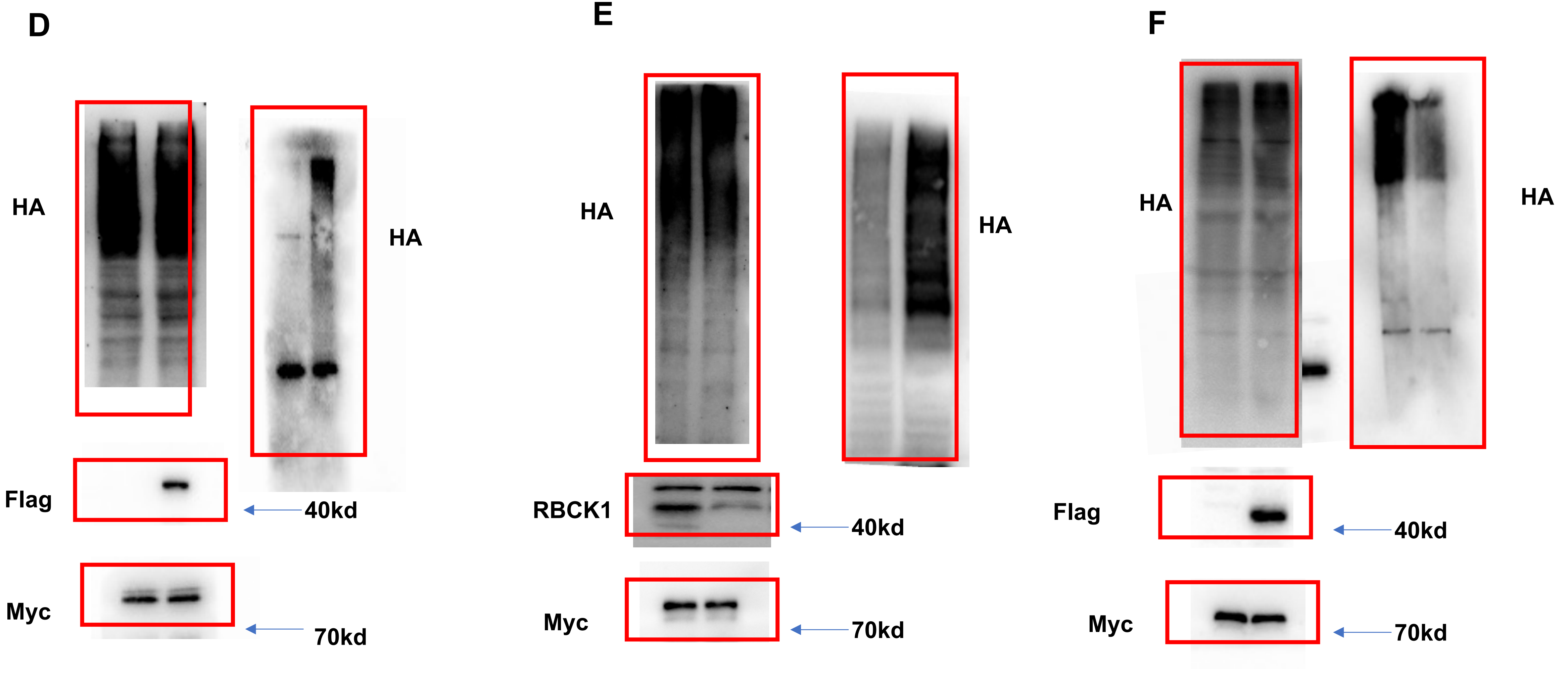
D
HA
HA
Flag
40kd
Myc
70kd
E
HA
HA
RBCK1
40kd
Myc
70kd
F
HA
HA
Flag
40kd
Myc
70kd

Figure 7

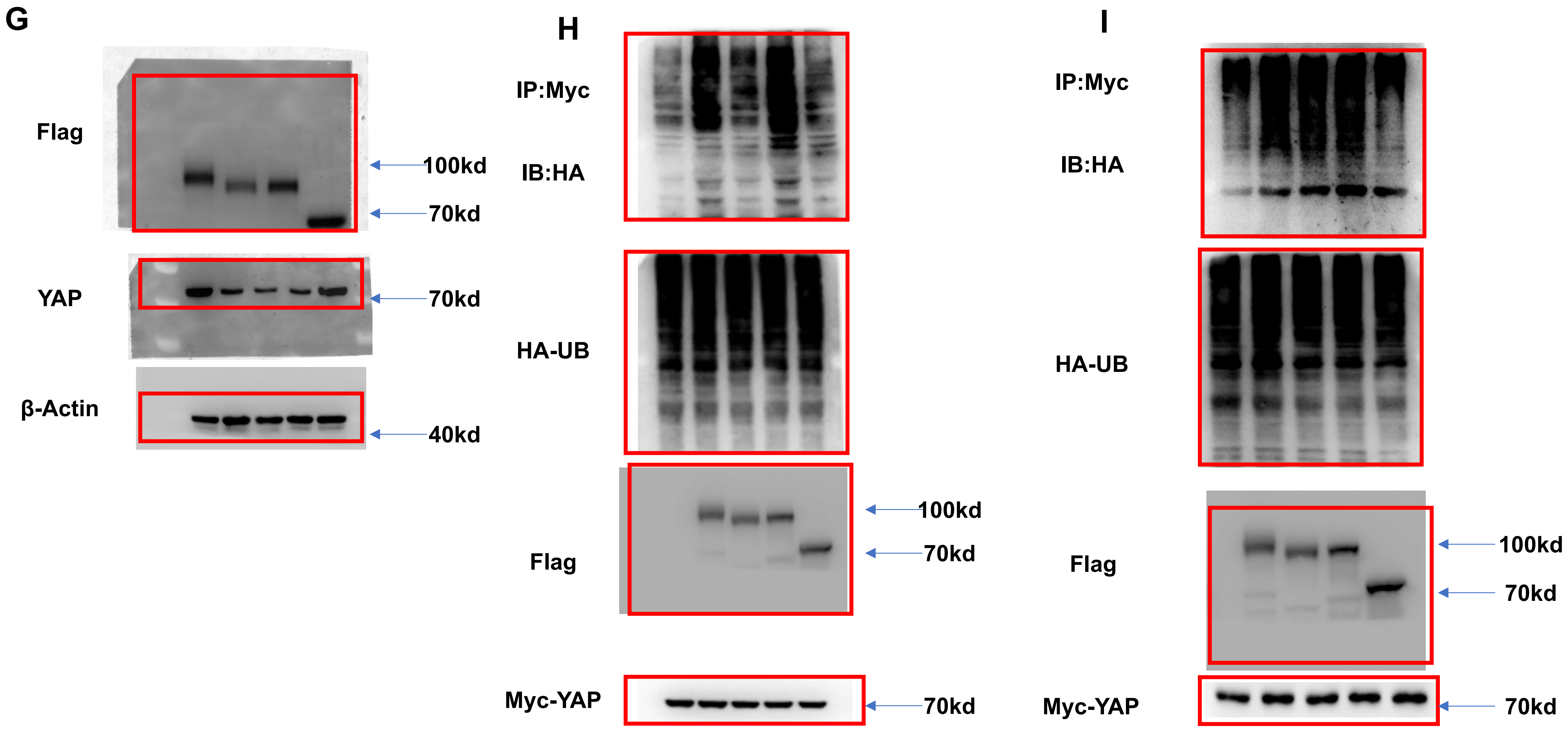
G
Flag
100kd
70kd
YAP
70kd
β-Actin
40kd
H
IP:Myc
IB:HA
HA-UB
Flag
100kd
70kd
Myc-YAP
70kd
I
IP:Myc
IB:HA
HA-UB
Flag
100kd
70kd
Myc-YAP
70kd

Figure 7

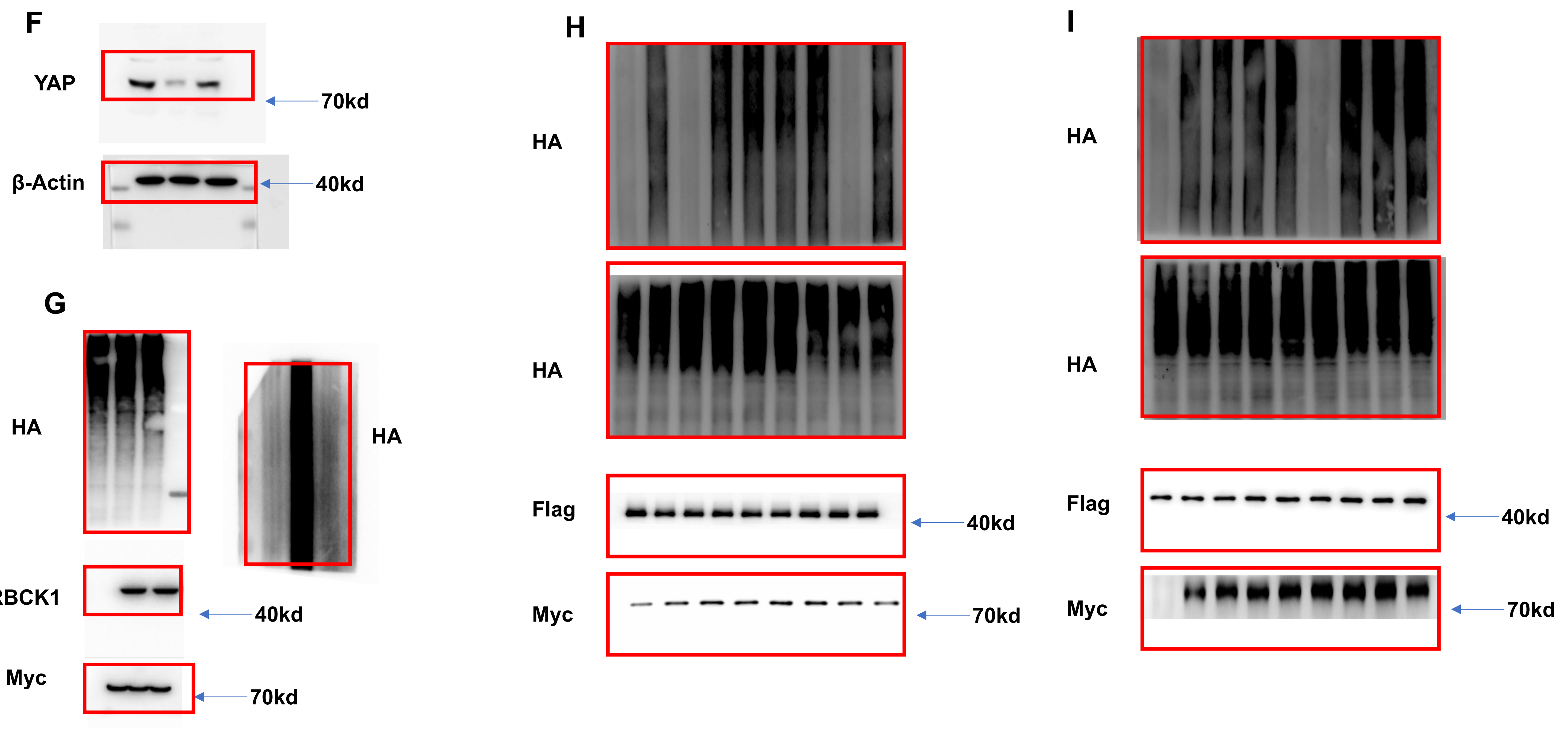

F
YAP
70kd
β-Actin
40kd
G
HA
HA
RBCK1
40kd
Myc
70kd
H
HA
HA
Flag
40kd
Myc
70kd
I
HA
HA
Flag
40kd
Myc
70kd
